# Activity of Vsr endonucleases encoded by *Neisseria gonorrhoeae* FA1090 is influenced by MutL and MutS proteins

**DOI:** 10.1186/s12866-018-1243-3

**Published:** 2018-08-30

**Authors:** Monika Adamczyk-Popławska, Katarzyna Bandyra, Agnieszka Kwiatek

**Affiliations:** Department of Virology, Institute of Microbiology, Faculty of Biology, University of Warsaw, Miecznikowa 1, 02-096 Warsaw, Poland

**Keywords:** *Neisseria gonorrhoeae*, DNA repair, Protein interactions, Vsr endonuclease, MutL protein

## Abstract

**Background:**

The functioning of DNA repair systems is based on correct interactions between proteins involved in DNA repair. Very Short Patch (VSP) repair is a DNA repair system that corrects mismatches resulting from the deamination of 5-methylcytosine. The key enzyme in the VSP system is Vsr endonuclease, which can cleave mismatched DNA independently of accessory proteins. Until now, in vivo activity has only been shown for V.EcoKDcm - the only Vsr endonuclease in *Escherichia coli.* Additionally, the VSP system of *E. coli* is the only one for which interactions between proteins of the system have been demonstrated. *Neisseria gonorrhoeae* FA1090 is the first bacterium that we previously demonstrated to encode two active in vitro Vsr endonucleases: V.NgoAXIII and V.NgoAXIV.

**Results:**

We elucidate the mutator phenotype of *N. gonorrhoeae* mutants with disrupted genes encoding V.NgoAXIII or V.NgoAXIV endonuclease. Furthermore, we investigate the interactions between gonococcal Vsr endonucleases and MutL and MutS proteins. The Vsr endonucleases physically interact with gonococcal MutL protein but not with MutS protein. In the presence of the MutL protein, the efficiency of DNA cleavage by both V.NgoAXIII and V.NgoAXIV endonucleases increases, resulting in a decrease in the amount of Vsr enzyme required to complete digestion of mismatched DNA. Both Vsr endonucleases are also stimulated in vitro by the MutL protein of *E. coli*. In turn, the gonococcal MutS protein hinders DNA cleavage by the Vsr endonucleases. However, this effect is overridden in the presence of MutL, and furthermore, the simultaneous presence of MutL and MutS causes an increase in the efficiency of DNA cleavage by the Vsr endonucleases compared to the reaction catalyzed by V.NgoAXIII or V.NgoAXIV alone.

**Conclusions:**

For the first time, interactions between proteins of the DNA repair system encoded by *N. gonorrhoeae* that are responsible for the correction of mismatches resulting from the 5-methylcytosine deamination were identified. The increase in activity of Vsr endonucleases in the presence of MutL protein could allow for reduced synthesis of the Vsr endonucleases in cells, and the susceptibility of gonococcal Vsr endonucleases on MutL protein of *E. coli* implies a universal mechanism of Vsr stimulation by MutL protein.

**Electronic supplementary material:**

The online version of this article (10.1186/s12866-018-1243-3) contains supplementary material, which is available to authorized users.

## Background

Many bacteria use 5-methylcytosine (m5C), which is produced by DNA C^5^-methyltransferases (C5MTases), to distinguish between self and non-self DNA and to regulate gene expression [1]. However, being less stable than unmethylated cytosine, m5C can undergo spontaneous deamination to thymine, which cannot be removed by general repair mechanisms because this thymine is not recognized as erroneous in DNA. As a result, T:G mismatches arise and, if not corrected, lead to C→T transitions [2]. In *Escherichia coli* K-12 possessing one C5MTase, the mutagenic effects of spontaneous deamination of m5C are offset by the Very Short Patch (VSP) repair system. The key enzyme in the *E. coli* VSP system is a single Vsr endonuclease (V.EcoKDcm), which cleaves the mismatched DNA on the 5′-side of the mispaired thymine to allow its specific removal. The in vitro digestion of mismatched DNA by V.EcoKDcm endonuclease is affected by MutL and MutS proteins, which also take part in the post-replicative DNA mismatch repair path [3–5]. Furthermore, in *E. coli* K-12, mutations in *mutL* and *mutS* genes limit in vivo VSP repair; however, they do not completely impair it [5, 6]. In vivo, DNA ligase and DNA polymerase I are also involved in the *E. coli* VSP system [7, 8].

Apart from *E. coli* K-12 [9, 10], the in vitro endonucleolytic activity of Vsr towards T:G mismatch has been shown for enzymes of *Bacillus stearothermophilus* H3 [11], *Neisseria gonorrhoeae* FA1090 [12] and *Neisseria meningitidis* [13]. Nonetheless, the *E. coli* K-12 Vsr endonuclease remains the only one for which the in vivo activity has been demonstrated and for which the physical and functional interactions with accessory proteins have been experimentally demonstrated [3, 8, 14].

*N. gonorrhoeae* FA1090 encodes five active C5MTases [15] that catalyze the creation of m5C. This bacterium is also the first and, thus far, the only microorganism shown to possess two in vitro active, phylogenetically-distant Vsr endonucleases, V.NgoAXIII and V.NgoAXIV. In contrast to the monospecific Vsr endonuclease of *E. coli* K-12, both gonococcal Vsr are multispecific. The former enzyme recognizes T:G mismatch in all nucleotide contexts, while the latter recognizes mismatches only in specific ones [12]. In addition to two Vsr endonucleases, *N. gonorrhoeae* FA1090 possesses one *mutL* and one *mutS* gene encoding proteins engaged in DNA repair [16]. However, it should be noted that unlike to *E. coli* K-12, our research model *N. gonorrhoeae* strain FA1090 does not encode MutH protein and Dam methyltransferase [17]. Moreover, many neisserial DNA repair pathways diverge from those of *E. coli* [18, 19].

The aim of this work was to determine the in vivo activity of the gonococcal Vsr endonucleases and to examine the presence and potential effects of interactions between the gonococcal Vsr enzymes and accessory proteins - MutL and MutS. We show that inactivation of *vsr* genes results in a mutator phenotype and that the activities of both types of gonococcal Vsr endonucleases are affected by both MutL and MutS proteins.

## Results

### Inactivation of *ngoAXIIIV*, *ngoAXIVV*, *mutL* and *mutS* genes results in a mutator phenotype of *N. gonorrhoeae*

The in vitro endonucleolytic activity of V.NgoAXIII and V.NgoAXIV protein towards DNA carrying a T:G mismatch [12] has been previously demonstrated. To study the in vivo activity of gonococcal Vsr endonucleases, gonococcal mutants were constructed by the gene replacement method [20]. To achieve this purpose, appropriate plasmids (Additional file 1: Table S2) were created and introduced into gonococcal cells to yield *N. gonorrhoeae ngoAXIIIV::km*, *N. gonorrhoeae ngoAXIVV::km*, *N. gonorrhoeae mutL::km* and *N. gonorrhoeae mutS::km* and complemented mutants.

**Fig. 1:**
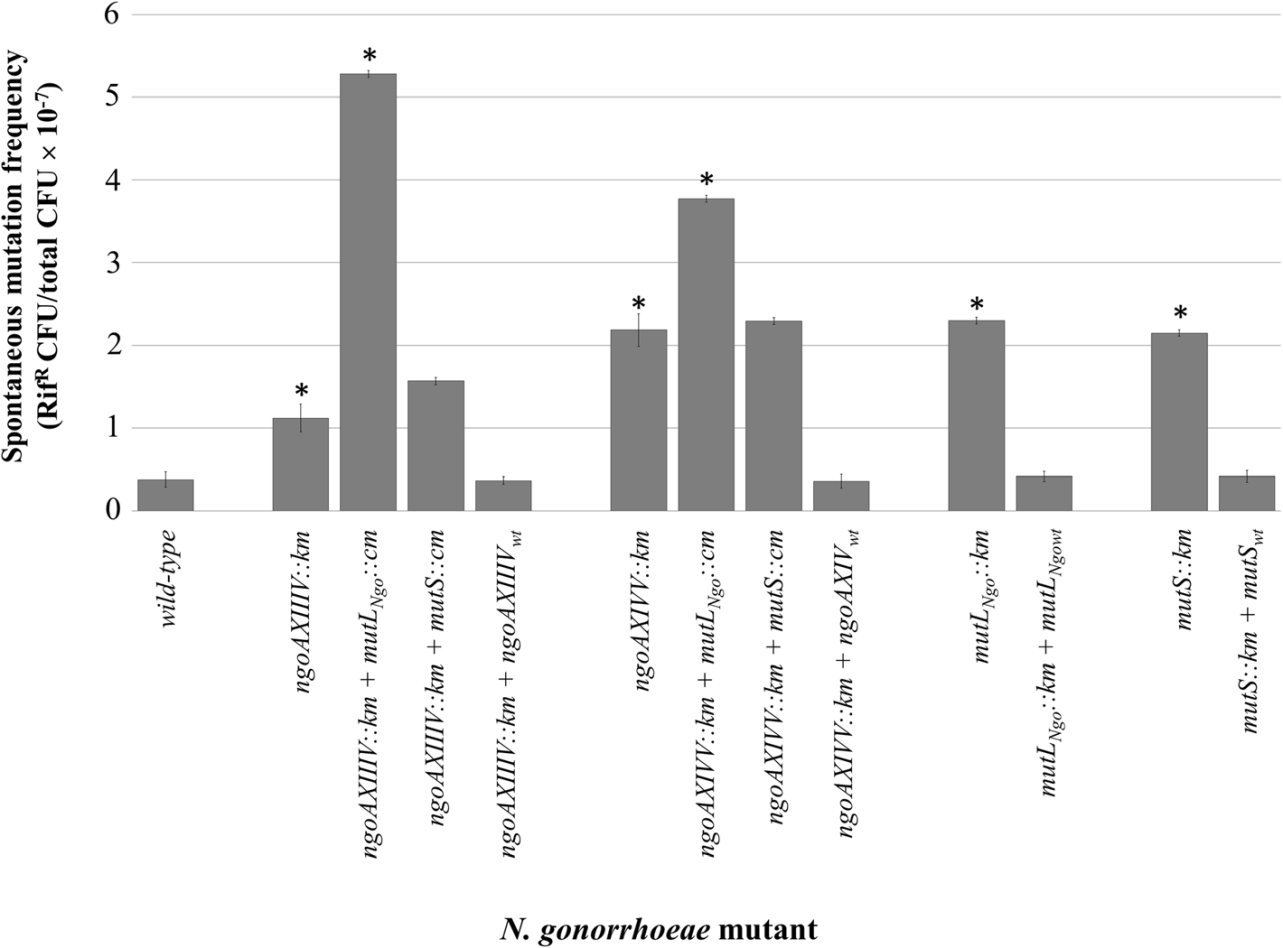
Spontaneous mutation frequency in *N. gonorrhoeae* mutants with disrupted *ngoAXIIIV*, *ngoAXIVV*, *mutL*_*Ngo*_ or *mutS* genes. A sample of liquid culture (0.1 ml, 10^8^ cells) of each strain was plated on GC agar supplemented with rifampicin or without antibiotics. After incubation at 37 °C in 5% CO_2_ for 48 h, the colony numbers were counted and frequency of spontaneous mutations causing rifampicin resistance determined. Asterisks indicate statistically significant differences compared to wild-type strain, which were calculated using the two-tailed heteroscedastic Student’s *t*-test (*p* value < 0.05)

Next, the frequency of spontaneous mutations in the obtained gonococcal mutant strains was assessed by screening for rifampicin [21–23] or nalidixic acid [16] resistance and compared with that of the parental wild-type strain FA1090. The strains lacking the individual Vsr endonucleases, MutL or MutS proteins showed elevated levels of spontaneous mutations upon rifampicin selection: the mutation frequency for the *ngoAXIIIV::km* strain was increased 2.97-fold, and those of the *ngoAXIVV::km*, *mutL::km*, *mutS::km* strains by 5.8-fold, 6.1-fold, and 5.7-fold, respectively, compared to the mutation frequency of the wild-type strain FA1090 (Fig. 1). The increase in mutation frequency was also observed upon nalidixic acid selection, by 3.5-fold, 4.65-fold, 5.2-fold and 6.7-fold, respectively, for gonococcal mutants with a disrupted *ngoAXIIIV, ngoAXIVV, mutL or mutS* gene (Additional file 1: Figure S1)*.* Following mutant complementation by in cis insertion of intact copies of the respective gene in the mutant strain chromosome, the frequency of spontaneous mutations, both upon rifampicin and nalidixic acid selection, returned to the wild-type level (Fig. 1, Additional file 1: Figure S1).

For *ngoAXIIIV::km* and *ngoAXIVV::km* mutant strains, sequencing of the central conserved region of the *rpoB* gene of gonococcal rifampicin-resistant colonies confirmed the appearance of the C→T transitions. Additionally, the transversions G→T, C→A and A→T, but no deletion and insertion mutations, were identified (Additional file 1: Table S4).

We also noted that inactivation of the *mutL* gene in *N. gonorrhoeae ngoAXIIIV::km* or *ngoAXIVV::km* mutants increased the frequency of spontaneous mutations, respectively, 14-fold and 10-fold compared to the wild-type strain FA1090. In contrast, the frequency of spontaneous mutations in the mutants with disrupted *mutS* and *ngoAXIIIV* or *mutS* and *ngoAXIVV* genes was not significantly different compared to mutants with a single gene disruption (Fig. 1).

These observations confirm the involvement of gonococcal Vsr endonucleases in DNA repair in vivo and also may suggest an association between MutL and gonococcal Vsr endonucleases.

### Gonococcal Vsr endonucleases physically interact with MutL but not with the MutS protein

The increase in mutation frequency in gonococcal mutants with disrupted *ngoAXIIIV* and *mutL* or *ngoAXIVV* and *mutL* genes led us to examine whether the MutL protein influences Vsr endonuclease activity via direct protein-protein interactions.

**Fig. 2:**
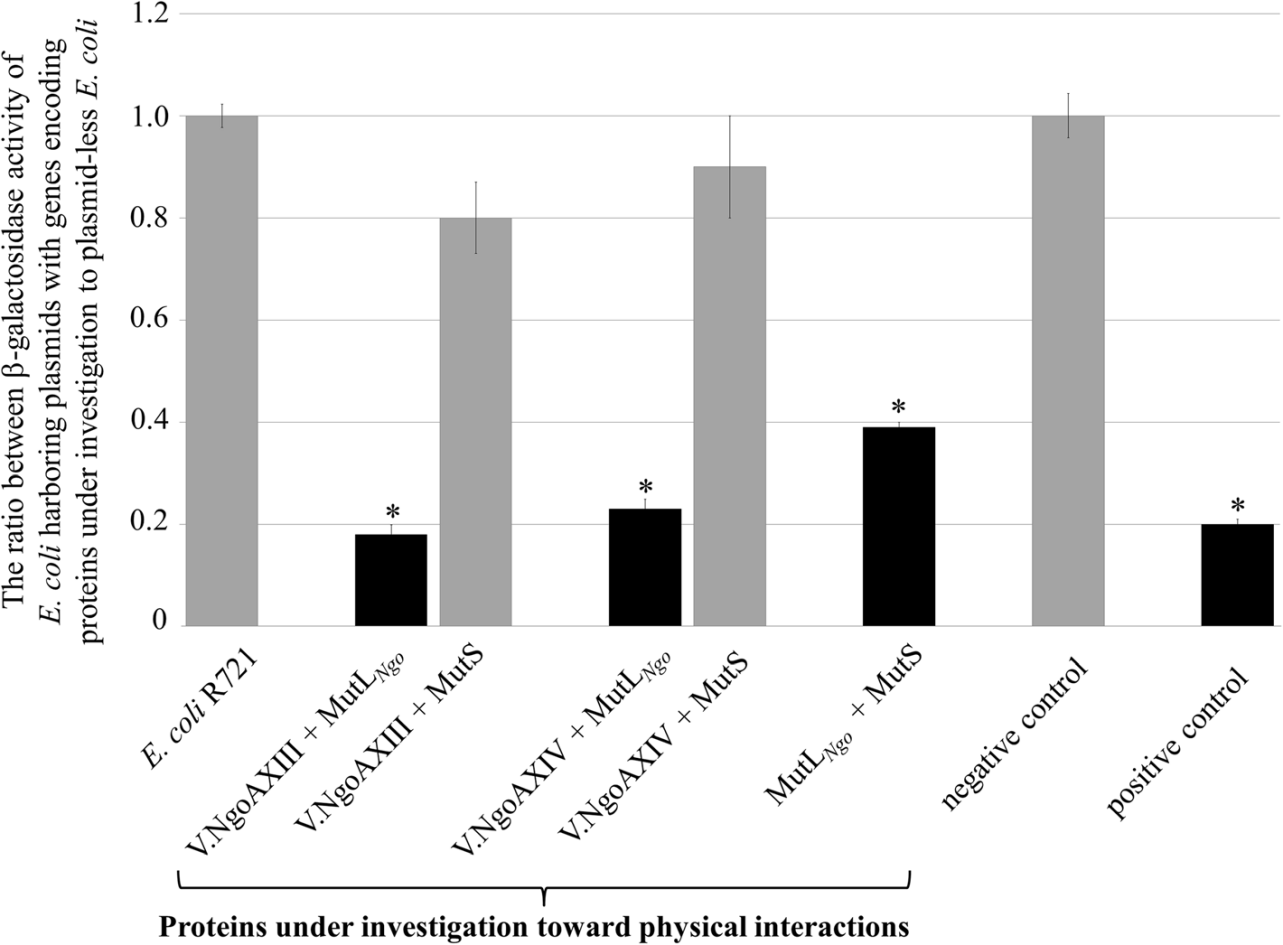
The interactions between Vsr endonucleases, MutL and MutS proteins of *N*. *gonorrhoeae* FA1090. The study was carried out using the bacterial two-hybrid system with *lacZ* as a reporter gene as described in the Methods section. The β-galactosidase activity was estimated and presented as Miller Units, and the ratio between *E. coli* cells harboring plasmids with genes encoding proteins under investigation to plasmid-less *E. coli* was calculated. Black bars indicate the presence of interactions between the investigated proteins. The results are the mean values obtained from at least six independent repeats. Asterisks indicate statistically significant differences, calculated using the two-tailed heteroscedastic Student’s *t*-test (*p* value < 0.05)

First, the physical interactions between the V.NgoAXIII and V.NgoAXIV proteins and MutL and MutS proteins were studied using a bacterial two-hybrid system with *lacZ* as a reporter gene. The interactions between the investigated proteins were determined based on the value of the Miller Units of the β-galactosidase activity ratio calculated for each pair of proteins. If the two proteins interacted with each other, the ratio was less than 0.5 (Fig. 2).

The ratio obtained for the *E. coli* R721 strain harboring plasmids with the genes *ngoAXIIIV* and *mutL*_*Ngo*_ or *ngoAXIVV* and *mutL*_*Ngo*_ to that of the plasmid-less one was 0.18 (±0.019) and 0.23 (±0.02), respectively. This finding indicated a physical interaction between the V.NgoAXIII or V.NgoAXIV endonuclease and the gonococcal MutL protein (MutL_*Ngo*_) (Fig. 2). The MutL_*Ngo*_ protein also interacted with MutS protein, as demonstrated by the ratio of Miller Units for *E. coli* R721 carrying plasmids with the *mutS* and *mutL*_*Ngo*_ genes to that of the plasmid-less strain of 0.39 (±0.01) (Fig. 2). In turn, the ratio of Miller Units for *E. coli* R721 harboring plasmids with the genes *mutS* and *ngoAXIIIV* or *mutS* and *ngoAXIVV* to that of the plasmid-less strain was 0.8 (±0.07) and 0.9 (±0.1), respectively (Fig. 2). This finding suggested that the MutS protein interacted neither with V.NgoAXIII nor V.NgoAXIV endonuclease. In the negative control, the ratio of Miller Units for *E. coli* R721 harboring a single plasmid to that of the plasmid-less strain was ~ 1.0 (±0.046), whereas in the positive control it was ~ 0.2 (±0.001).

**Fig. 3:**
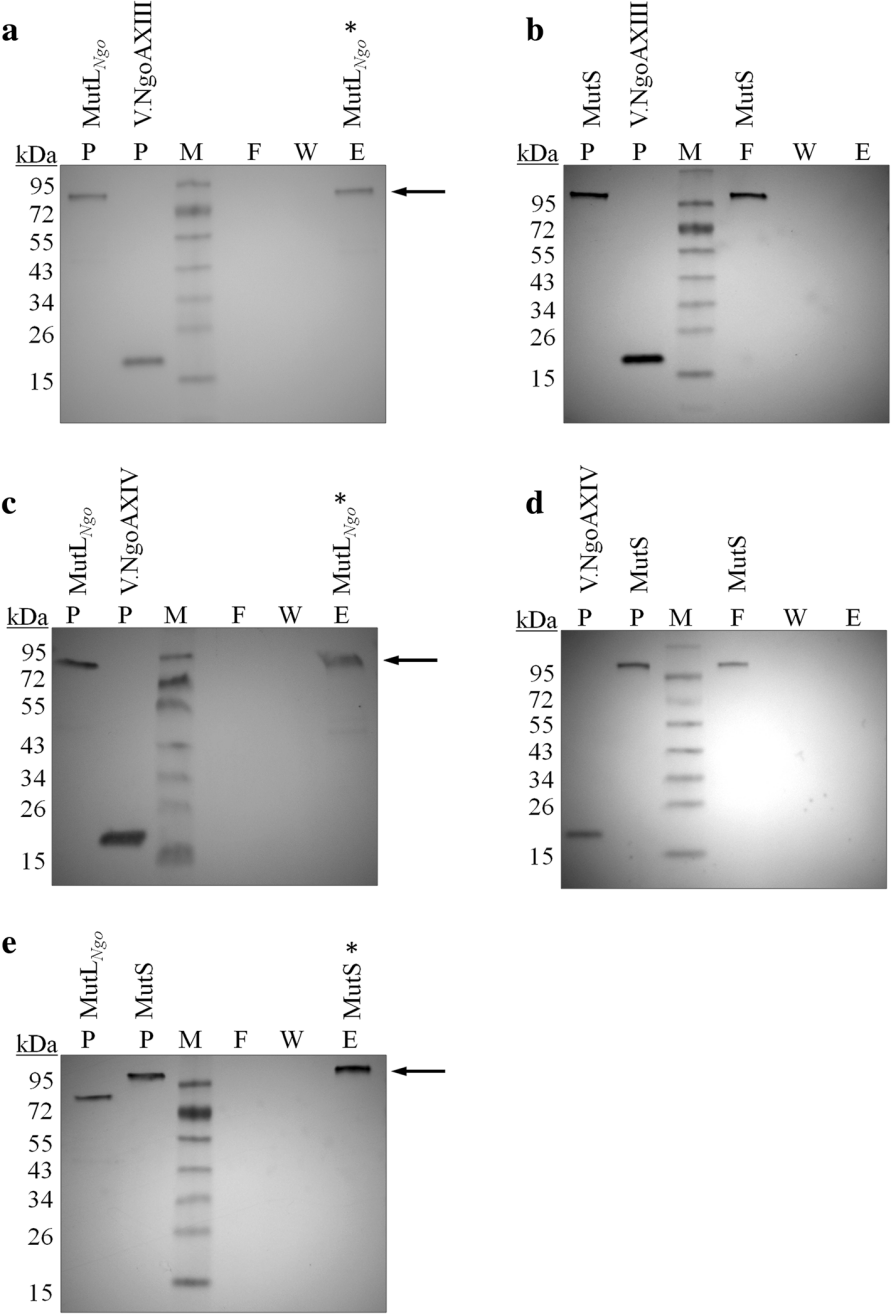
Interactions between the gonococcal Vsr endonucleases, MutL and MutS proteins studied by protein affinity chromatography. Analysis of the interactions between (**a**) V.NgoAXIII and MutL_*Ngo*_; **b** V.NgoAXIII and MutS; **c** V.NgoAXIV and MutL_*Ngo*_; **d** V.NgoAXIV and MutS; and (**e**) MutS and MutL_*Ngo*_*.* V.NgoAXIII, V.NgoAXIV or MutL_*Ngo*_ proteins were coupled to affinity gel as ligands, and subsequently His-tagged MutS or MutL_*Ngo*_ were applied to the resin. Representative Western blots with antibodies against His-Tag are presented. The arrows and asterisks indicate protein only in the elution fraction. P – purified proteins used in the experiment; F – flow through fraction; W - wash fraction; E - elution fraction; M - PageRuler™ Prestained Protein Ladder (250, 130, 95, 72, 55, 43, 34, 26, 15 and 11 kDa) (Thermo Scientific)

The in vitro interactions between purified Vsr endonucleases and MutL_*Ngo*_ protein, Vsr endonucleases and MutS protein and MutL_*Ngo*_ and MutS proteins were studied by protein affinity chromatography. As is shown in Fig. 3, MutL_*Ngo*_ protein was visible only in the elution but not in flow-through or wash fractions, after using resin with V.NgoAXIII or V.NgoAXIV endonucleases as ligand. This result indicated that the MutL_*Ngo*_ protein was retained on the Vsr affinity column. Additionally, MutS protein was visible only in the elution but not in flow-through and wash fractions after using resin with MutL_*Ngo*_ as ligand, supporting an interaction between these proteins.

In turn, MutS protein was in the flow-through and wash fractions when resin with V.NgoAXIII or V.NgoAXIV protein was used as ligand (Fig. 3), which indicated that MutS was not retained by endonucleases. In summary, these results support the conclusion that physical interactions occur between MutL_*Ngo*_ protein and V.NgoAXIII, MutL_*Ngo*_ and V.NgoAXIV and between MutL_*Ngo*_ and MutS protein, but not between MutS protein and Vsr endonucleases.

### The activity of both gonococcal Vsr endonucleases is stimulated by the gonococcal MutL protein

Previously, we have demonstrated the endonucleolytic activity of the gonococcal Vsr endonucleases in the absence of any accessory proteins. However, to complete digestion of the substrate DNA, a 10-fold excess of enzyme over DNA is required [12]. The occurrence of interactions between gonococcal Vsr endonucleases and the MutL_*Ngo*_ protein prompted us to examine whether physical interactions influence the in vitro activity of V.NgoAXIII or V.NgoAXIV endonucleases. For this purpose, the first order rate constant (*k*_st_) of the reactions catalyzed by the Vsr endonucleases in the presence of MutL_*Ngo*_ protein and in the absence of MutS was estimated using a two-fold excess Vsr endonuclease over substrate DNA. Since it is assumed that Vsr enzymes act as monomers [24] and bacterial MutL proteins are functional homodimers [25, 26], DNA cleavage by Vsr endonucleases in the presence of MutL_*Ngo*_ was studied using a molar ratio purified proteins of 1:2 (Vsr:MutL_*Ngo*_). Additionally, as ATP binding is required to instigate conformational changes in the MutL domain responsible for the regulation of interactions of MutL with other components in the cellular DNA repair machinery [25, 26], each of the above reactions was carried out in two variants: in the presence of 1 mM ATP or without supplementation with ATP. The obtained data were compared to *k*_st_ of the reaction catalyzed by Vsr endonucleases without MutL and ATP.

**Fig. 4:**
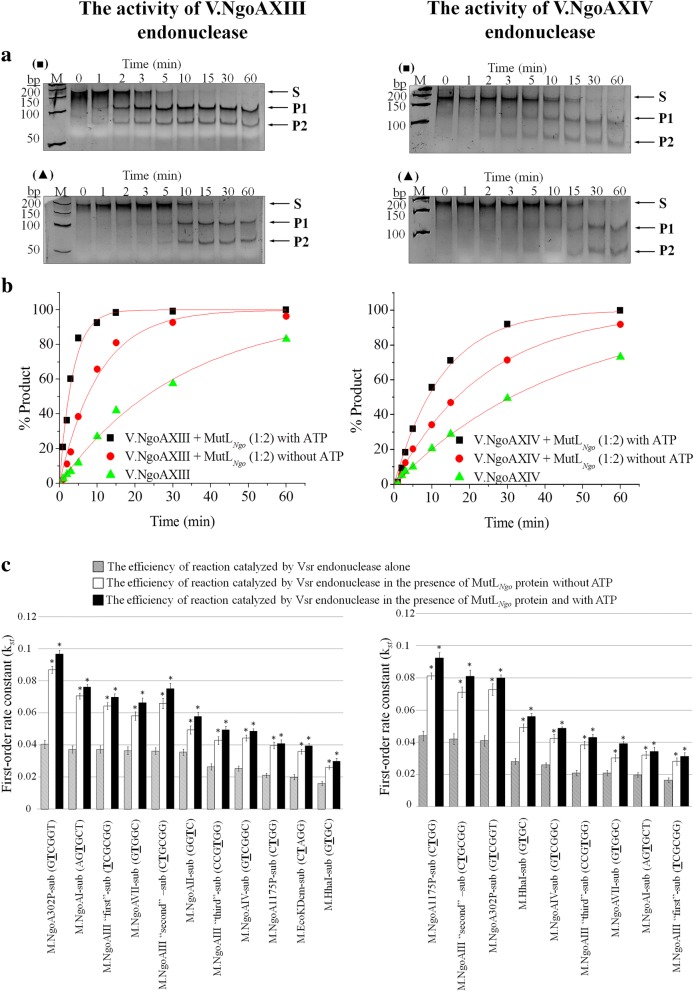
The activity of the gonococcal Vsr endonucleases in the presence of gonococcal MutL protein. Left part of the figure – the activity of V.NgoAXIII; right part of the figure – the activity of V.NgoAXIV endonuclease. **a** Examples of electrophoresis profiles of the reaction products resolved in a 10% polyacrylamide gel: (black squares) DNA cleavage by gonococcal Vsr endonuclease in the absence of both MutL_*Ngo*_ protein and 1 mM ATP; (black triangles) DNA cleavage by gonococcal Vsr endonuclease in the presence of MutL_*Ngo*_ protein and 1 mM ATP, molar ratio Vsr:MutL 1:2. These and analogous profiles were used to quantify the efficiency of cleavage of substrate DNAs containing a T:G mismatch, catalyzed by Vsr endonucleases. M – marker GeneRuler 50 bp DNA Ladder (Thermo Scientific). Arrows indicate substrate and reaction products obtained after DNA cleavage by a given Vsr endonuclease. P1 and P2 reaction products; S – substrate DNA. **b** Analysis of the data obtained and fitted to a first-order rate equation using Origin 8.5 software. The presented results were obtained using M.NgoA302P-sub (GTCGGT/ACCGGC) for V.NgoAXIII and M.NgoA1175P-sub (CTGG/CCGG) substrate DNA for V.NgoAXIV. **c** Histograms presenting first-order rate constants (*k*_*st*_) obtained for endonucleolytic reactions catalyzed by gonococcal Vsr endonucleases in the presence and absence of the gonococcal MutL_*Ngo*_ protein. Asterisks indicate statistically significant differences, which were calculated using the two-tailed heteroscedastic Student’s *t*-test (*p* value < 0.05). Reactions were carried out using 0.15 μM substrate DNA, 0.3 μM Vsr endonuclease and 0.6 μM MutL_*Ngo*_ protein. The experiments were performed in triplicate, and representative electrophoresis profiles and graphs of the data fitted to a first-order rate equation are shown

The V.NgoAXIII endonuclease alone most efficiently cleaves M.NgoA302P-sub substrate (GTTCGGT/ACCGGC) [12]. The efficiency of cleavage of this substrate by V.NgoAXIII in the presence of MutL_*Ngo*_ and 1 mM ATP increased by 138.7% (*k*_st_ value 0.0967) in comparison to the control reaction carried out by the endonuclease alone (*k*_st_ = 0.0405) (Fig. 4). Since V.NgoAXIII is multispecific, the influence of the MutL_*Ngo*_ protein on the DNA cleavage efficiency of all 11 substrates (Additional file 1: Table S3) recognized by Vsr was tested. As presented in Fig. 4 and Additional file 1: Table S5A, a similar effect as for M.NgoA302P-sub substrate of the MutL_*Ngo*_ protein on DNA digestion by V.NgoAXIII was observed for all other tested DNAs. The efficiency of digestion of these DNAs by V.NgoAXIII increased by 62.6–107.1% (*k*_st_ = 0.0298–0.0762) compared to the control (*k*_st_ = 0.0160–0.0373).

In the absence of ATP, the level of stimulation of the V.NgoAXIII activity by MutL_*Ngo*_ was reduced. Nonetheless, for all substrates, *k*_st_ for the variants with MutL_*Ngo*_ still remained higher compared with the reaction with endonuclease alone (Fig. 4, Additional file 1: Table S5A).

An analogous set of experiments was performed for the V.NgoAXIV endonuclease, and the obtained results were similar to those for V.NgoAXIII. For all nine substrates recognized by V.NgoAXIV, the highest Vsr cleavage rates were observed in the reactions in the presence of the MutL_*Ngo*_ protein and 1 mM ATP. In such an experimental variant, cleavage efficiency for different substrates increased by 73.5–110% (*k*_st_ = 0.0313–0.0924) compared to the control (*k*_st_ = 0.0165–0.0440) (Fig. 4; the results for M.NgoA1175P-sub (CTGG/CCGG) substrate cleavage are presented in the figure only, and other obtained *k*_st_ are presented in Fig. 4 and listed in Additional file 1: Table S5B). Without ATP, *k*_st_ values ranged between 0.0282 and 0.0812, depending on the substrate DNA, but they were still higher than those obtained for the reaction with the endonuclease alone *k*_st_ = 0.0165–0.0440 (Fig. 4, Additional file 1: Table S5B).

**Fig. 5:**
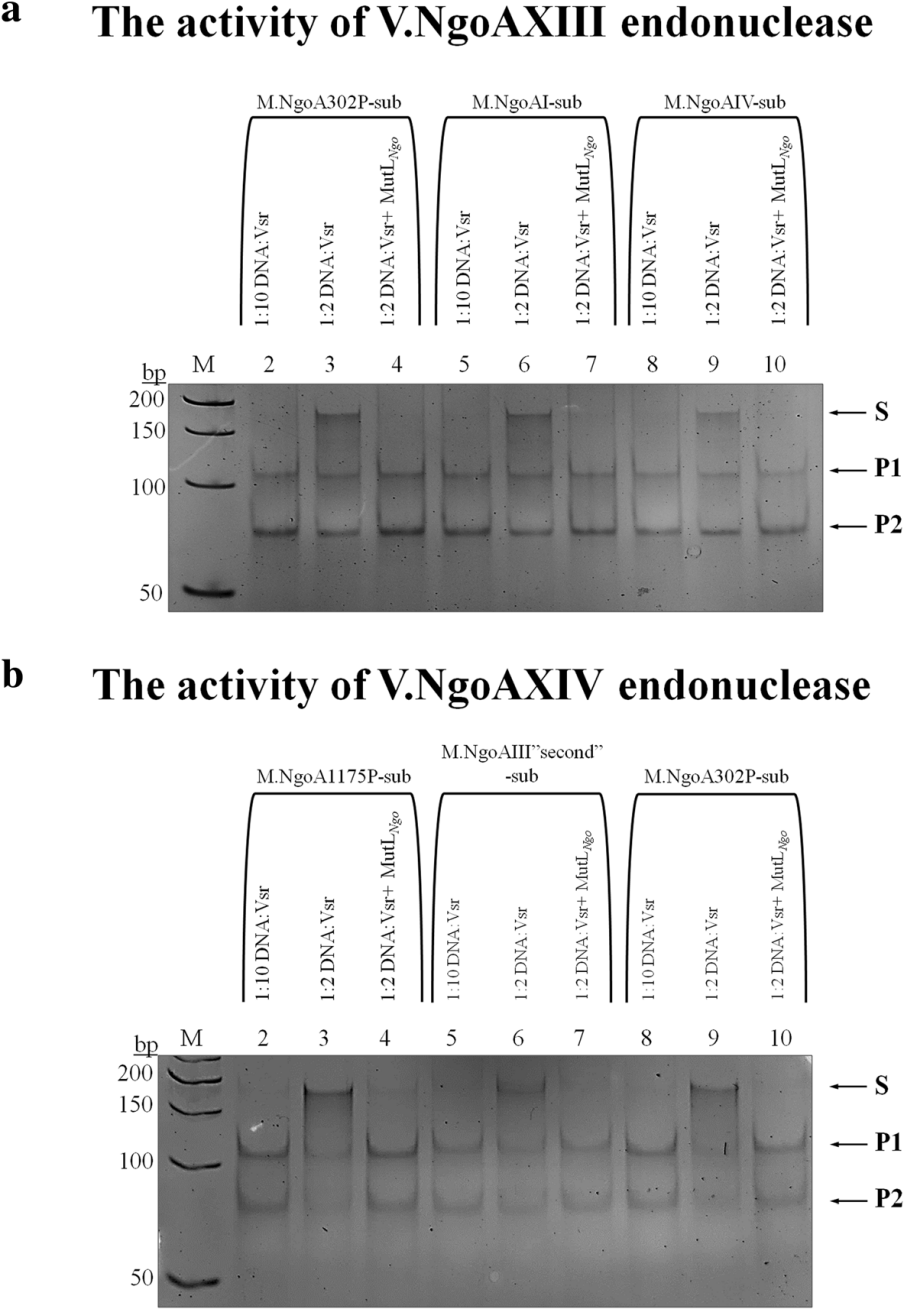
The presence of MutL_*Ngo*_ protein reduces the amount of Vsr endonuclease required for DNA digestion. The presented results were obtained (**a**) using M.NgoA302P-sub (GTCGGT/ACCGGGC) (lane 2, 3, 4), M.NgoAI-sub (AGTGCT/AGCGCT) (lane 5, 6, 7) and M.NgoAIV-sub (GTCGGC/GCCGGC) (lane 8, 9, 10) for V.NgoAXIII and (**b**) M.NgoA1175P-sub (CTGG/CCGG) (lane 2, 3, 4), M.NgoAIII “second”-sub (CTGCGG/CCGCGG) (lane 5, 6, 7) and M.NgoA302P-sub (GTCGGT/ACCGGC) (lane 8, 9, 10) for V.NgoAXIV. Reactions were carried out using 0.15 μM substrate DNA, 0.3 μM or 1.5 μM Vsr endonuclease and, where indicated, 0.6 μM MutL_*Ngo*_ protein. M – marker GeneRuler 50 bp DNA Ladder (Thermo Scientific). Arrows indicate substrate and reaction products obtained after DNA cleavage by a given Vsr endonuclease. P1 and P2 reaction products; S – substrate DNA. The experiments were performed in triplicate, and representative images are shown

Furthermore, as presented in Fig. 5, the increase in efficiency of DNA cleavage by V.NgoAXIII or V.NgoAXIV in the presence of the MutL_*Ngo*_ protein indicated that the two-fold excess of Vsr endonuclease over substrate DNA was sufficient to complete DNA digestion instead of 10-fold in the control reaction (without accessory MutL_*Ngo*_ protein). Such results were observed for all tested substrates for both V.NgoAXIII and V.NgoAXIV endonucleases (Fig. 5 and Additional file 1: Figure S2).

The efficiency of the control reactions carried out by the gonococcal Vsr endonucleases in the presence of 1 mM ATP or BSA was the same as in the reaction with Vsr endonucleases alone. No DNA cleavage products were observed when the MutL_*Ngo*_ alone was incubated with different substrate DNAs.

To conclude, under in vitro conditions, the gonococcal MutL protein significantly increased the efficiency of the reaction catalyzed by the gonococcal Vsr endonucleases, and the presence of ATP enhanced this effect.

### The activity of gonococcal Vsr endonucleases is also stimulated by MutL protein of *E. coli* K-12

**Fig. 6:**
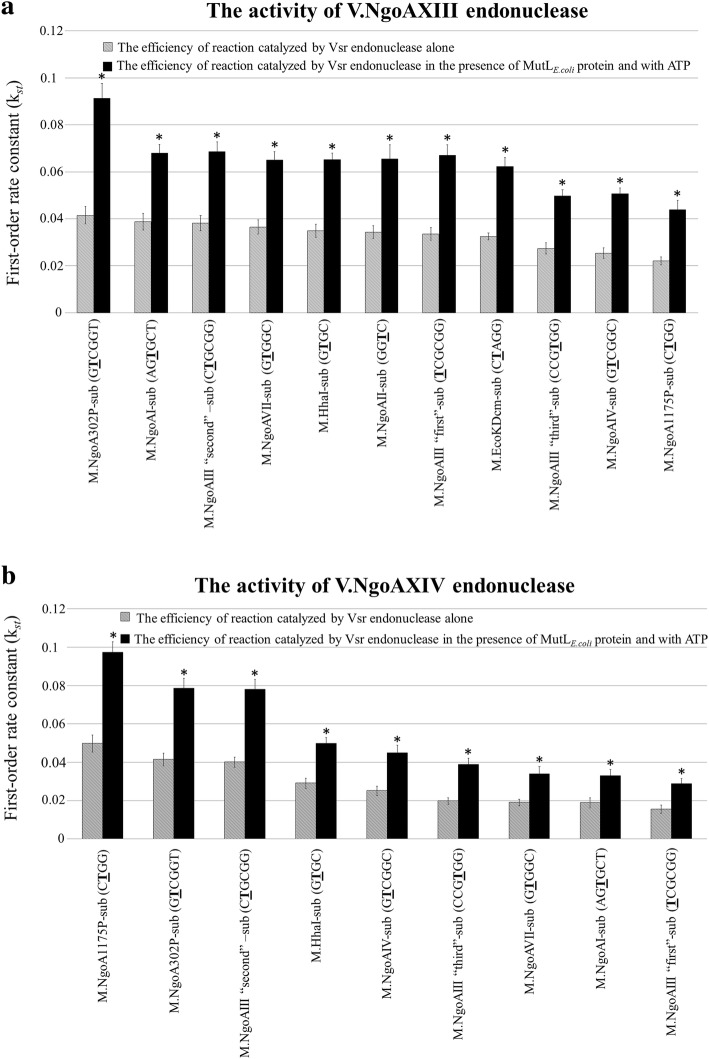
The activity of (**a**) V.NgoAXIII endonuclease and (**b**) V.NgoAXIV endonuclease in the presence of MutL_*E.coli*_ protein encoded by *E. coli*. Histograms presenting first-order rate constants (*k*_*st*_) obtained for endonucleolytic reactions catalyzed by gonococcal Vsr endonucleases in the presence and absence of MutL_*E.coli*_ protein. Reactions were carried out using 0.15 μM substrate DNA, 0.3 μM or 1.5 μM Vsr endonuclease and, where indicated, 0.6 μM MutL_*E.coli*_ protein, and first-order rate constants (*k*_*st*_) were determined. Asterisks indicate statistically significant differences, which were calculated using the two-tailed heteroscedastic Student’s *t*-test (*p* value < 0.05)

The high conservation of the N-terminal domain among proteins of the MutL superfamily and the 62% identity between N-terminal domains of MutL proteins from *N. gonorrhoeae* and *E. coli* led us to examine whether the in vitro activity of the gonococcal Vsr endonucleases could also be affected by the MutL protein of *E. coli* K-12 (MutL_*E.coli*_). For this purpose, the efficiency and rate of reactions carried out by V.NgoAXIII or V.NgoAXIV in the presence of purified MutL_*E.coli*_ at a molar ratio Vsr:MutL_*E.coli*_ 1:2 in the presence of 1 mM ATP were studied. Similar to the above-described experiments with the MutL_*Ngo*_ protein, the efficiency of DNA cleavage by V.NgoAXIII or V.NgoAXIV increased in the presence of MutL_*E.coli*_ for all the tested DNA substrates. The activity of V.NgoAXIII and V.NgoAXIV was higher by 75–120.1% (*k*_st_ = 0.0439–0.0913) and 72.3–98.7% (*k*_st_ = 0.0289–0.0974), respectively, compared to the reaction without MutL_*E.coli*_ and without ATP (*k*_st_ = 0.0221–0.0415 for V.NgoAXIII and 0.0155–0.0497 for V.NgoAXIV) (Fig. 6, Additional file 1: Table S6). In the control reaction with MutL_*E.coli*_ protein only (without Vsr endonucleases), no DNA cleavage products were observed.

In conclusion, the activity of the gonococcal V.NgoAXIII and V.NgoAXIV endonucleases may also be stimulated by MutL protein of *E. coli* K-12.

### Gonococcal MutS protein decreases the efficiency of the hydrolytic reactions catalyzed by gonococcal Vsr endonucleases

The potential impact of the MutS protein on the efficiency of DNA cleavage catalyzed by the V.NgoAXIII and V.NgoAXIV endonucleases was estimated based on the *k*_st_ values obtained for the reactions carried out in the presence of the MutS protein at a molar ratio of 1:2 and in the presence of 1 mM ATP. Similarly, as for the study concerning the influence of MutL proteins on Vsr endonuclease activity, the impact of MutS protein was also tested for all substrates recognized by V.NgoAXIII and V.NgoAXIV endonucleases.

**Fig. 7:**
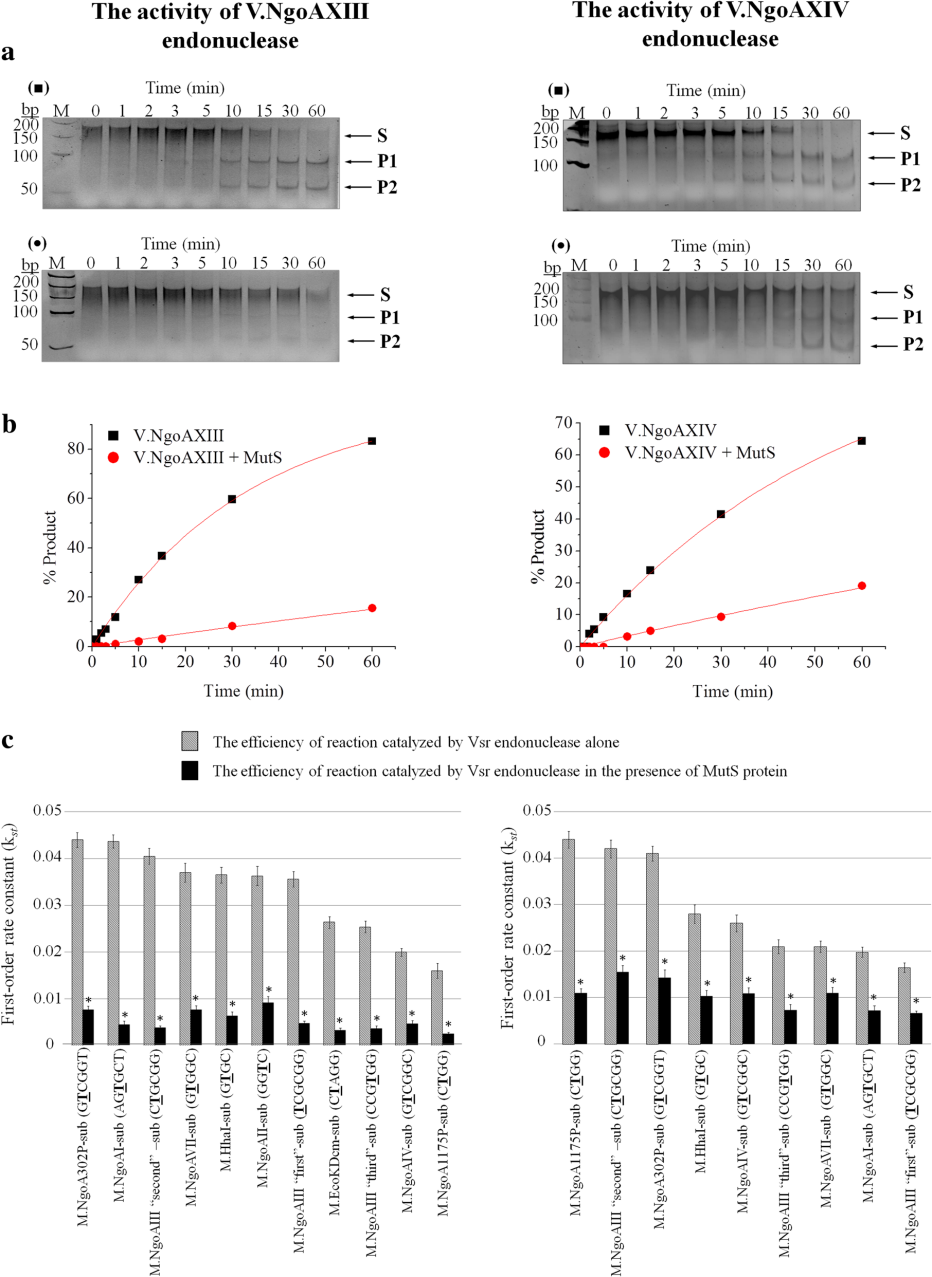
The influence of the MutS protein on DNA cleavage by V.NgoAXIII and V.NgoAXIV endonucleases. Left part of the figure – the activity of V.NgoAXIII; right part of the figure – the activity of the V.NgoAXIV endonuclease. **a** Examples of electrophoresis profiles of the reaction products resolved in a 10% polyacrylamide gel: (black squares) DNA cleavage by the gonococcal Vsr endonuclease in the absence of both MutS protein and 1 mM ATP; (black triangles) DNA cleavage by the gonococcal Vsr endonuclease in the presence of MutS protein and 1 mM ATP, molar ratio Vsr:MutS 1:2. These and analogous profiles were used to quantify the efficiency of cleavage of the substrate DNAs containing a T:G mismatch catalyzed by Vsr endonucleases. M - GeneRuler 50 bp DNA Ladder (Thermo Scientific). Arrows indicate substrate and reaction products obtained after DNA cleavage by a given Vsr endonuclease. P1 and P2 reaction products; S – substrate DNA. **b** Analysis of the data obtained and fitted to a first-order rate equation using Origin 8.5 software. The presented results were obtained using M.NgoA302P-sub (GTCGGT/ACCGGC) for V.NgoAXIII and M.NgoA1175P-sub (CTGG/CCGG) substrate DNA for V.NgoAXIV. **c** Histograms presenting first-order rate constants (*k*_*st*_) obtained for endonucleolytic reactions catalyzed by gonococcal Vsr endonucleases in the presence and absence of gonococcal MutS protein. Asterisks indicate statistically significant differences, which were calculated using the two-tailed heteroscedastic Student’s *t*-test (*p* value < 0.05). Reactions were carried out using 0.15 μM substrate DNA, 0.3 μM Vsr endonuclease and, where indicated, 0.6 μM MutS protein. The experiments were performed in triplicate, and representative images and graphs of the data fitted to a first-order rate equation are shown

The activity of the V.NgoAXIII endonuclease decreased in the presence of MutS and ATP for all tested substrates (Fig. 7, Additional file 1: Table S7A). This enzyme, in the presence of the MutS protein and 1 mM ATP, digested various substrate DNAs less efficiently, by 74.9–90.6% (*k*_st_ = 0.00246–0.0091), compared to the cleavage reaction performed in the absence of MutS protein (*k*_st_ = 0.0160–0.0440) (Fig. 7, Additional file 1: Table S7A).

Similar to the results for V.NgoAXIII, in the presence of MutS protein and 1 mM ATP, V.NgoAXIV endonuclease cleaved the substrate DNAs less efficiently, by 47.2–75% (*k*_st_ = 0.0066–0.0155), than in the absence of MutS (*k*_st_ = 0.0165–0.0440) (Fig. 7, Additional file 1: Table S7B).

In conclusion, the activity of both gonococcal Vsr endonucleases was significantly repressed by MutS protein towards substrates containing a T:G mismatch in all nucleotide sequence context.

### V.NgoAXIII and V.NgoAXIV endonuclease activity increases in the simultaneous presence of MutL_*Ngo*_ and MutS proteins

**Fig. 8:**
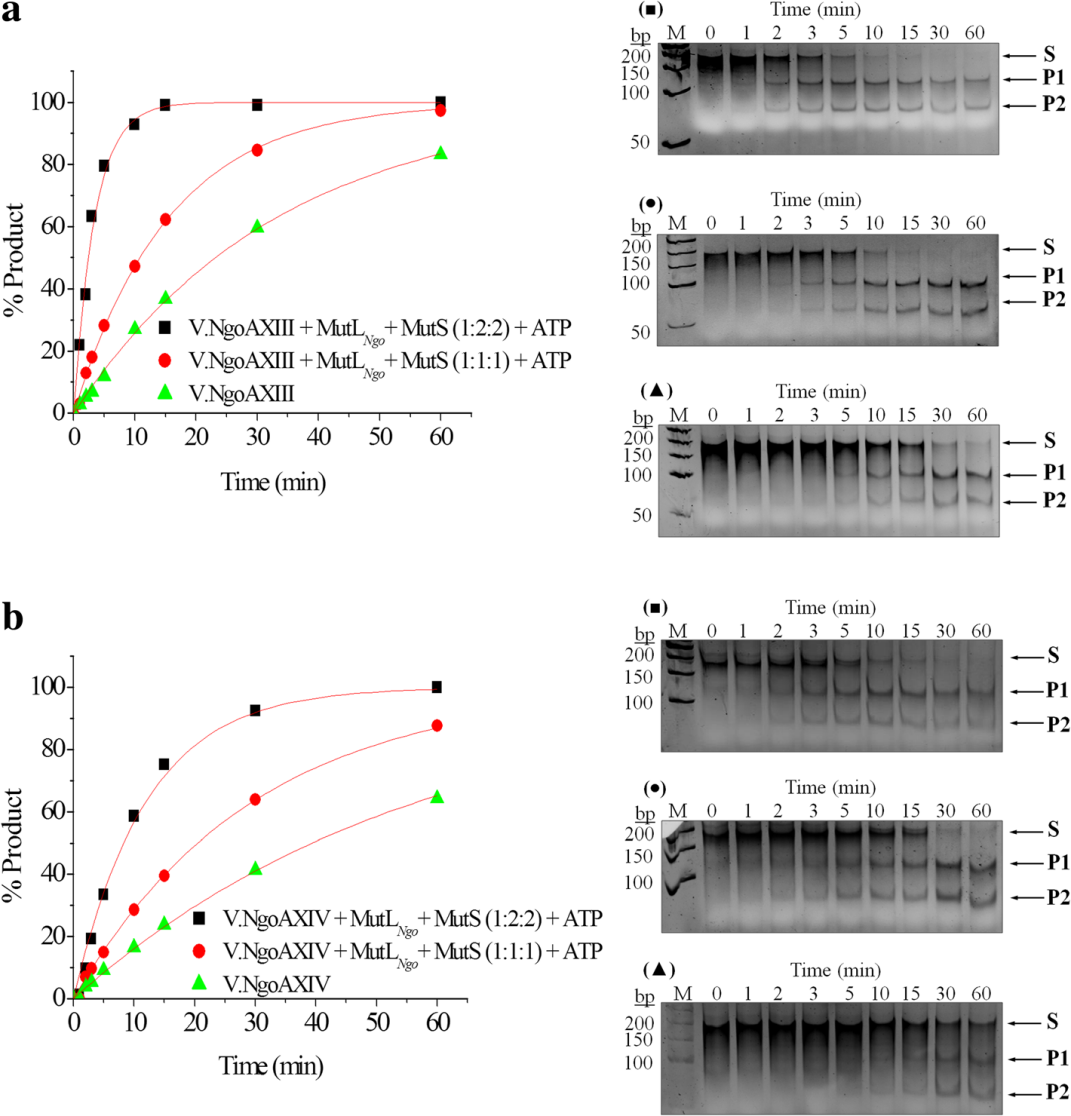
DNA cleavage by Vsr endonucleases in the simultaneous presence of MutL_*Ngo*_ and MutS proteins. The activity of (**a**) the V.NgoAXIII or (**b**) V.NgoAXIV endonucleases. The graphs show the data obtained and fitted to a first-order rate equation using Origin 8.5 software. Below the graphs are examples of the electrophoresis profiles of the reaction products resolved in a 10% polyacrylamide gel after digestion of substrate DNA by V.NgoAXIII or V.NgoAXIV: (black triangles) DNA cleavage by gonococcal Vsr endonuclease in the absence of MutL_*Ngo*_, MutS proteins and 1 mM ATP; (black circles) DNA cleavage by gonococcal Vsr endonuclease in the presence of MutL_*Ngo*_, MutS proteins, and 1 mM ATP, molar ratio Vsr:MutL:MutS 1:1:1; (black squares) DNA cleavage by gonococcal Vsr endonuclease in the presence of MutL_*Ngo*_, MutS proteins, and 1 mM ATP, molar ratio Vsr:MutL:MutS 1:2:2. M - GeneRuler 50 bp DNA Ladder (Thermo Scientific). The presented results were obtained using M.NgoA302P-sub (GTCGGT/ACCGGC) for V.NgoAXIII and M.NgoA1175P-sub (CTGG/CCGG) substrate DNA for V.NgoAXIV. Arrows indicate substrate and reaction products obtained after DNA cleavage by a given Vsr endonuclease. P1 and P2 reaction products; S – substrate DNA. The experiments were performed in triplicate, and representative images are shown

As observed, the efficiency of DNA cleavage catalyzed by V.NgoAXIII or V.NgoAXIV increased in the presence of the MutL_*Ngo*_ but decreased in the presence of MutS. Therefore, we decided to investigate the effect of the simultaneous presence of MutL_*Ngo*_ and MutS proteins on the activity of gonococcal Vsr endonucleases using M.NgoA302P-sub (GTCGGT/ACCGGC) and M.NgoA1175P-sub (CTGG/CCGG) substrates for V.NgoAXIII and V.NgoAXIV, respectively. In the presence of MutL_*Ngo*_ and MutS proteins, the efficiency of endonucleolytic reactions catalyzed by V.NgoAXIII endonuclease increased by 60.82% when the Vsr:MutL:MutS molar ratio was 1:1:1 (*k*_st_ = 0.0644) and by 139.3% (*k*_st_ = 0.0959) for the 1:2:2 variant, compared to the reaction carried out by the Vsr endonucleases alone (*k*_st_ = 0.0401) (Fig. 8a).

The V.NgoAXIV endonuclease in the simultaneous presence of MutL_*Ngo*_ and MutS proteins cleaved the substrate DNA with an efficiency increase of 41.01% (*k*_st_ = 0.0549) and 91.2% (*k*_st_ = 0.0746) for Vsr:MutL:MutS molar ratio variants of 1:1:1 and 1:2:2, respectively, compared to the reaction carried out by the Vsr endonucleases alone (*k*_st_ = 0.039) (Fig. 8b).

In summary, the reaction rate of DNA cleavage by V.NgoAXIII and V.NgoAXIV was enhanced in the simultaneous presence of MutL, MutS and ATP compared to Vsr endonucleases alone.

### DNA binding by V.NgoAXIII and V.NgoAXIV endonucleases and MutS protein

As demonstrated above, the presence of MutS decreased the efficiency of the mismatched DNA cleavage by V.NgoAXIII and V.NgoAXIV endonucleases. This effect may be a result of the DNA binding capabilities of these proteins toward the same substrate DNA carrying a T:G mismatch. To test this hypothesis, DNA binding by the studied proteins was estimated in the presence or absence of 1 mM ATP using M.NgoA302P-sub (GTCGGT/ACCGGC) and M.NgoA1175P-sub (CTGG/CCGG) substrates for V.NgoAXIII and V.NgoAXIV, respectively.

**Fig. 9:**
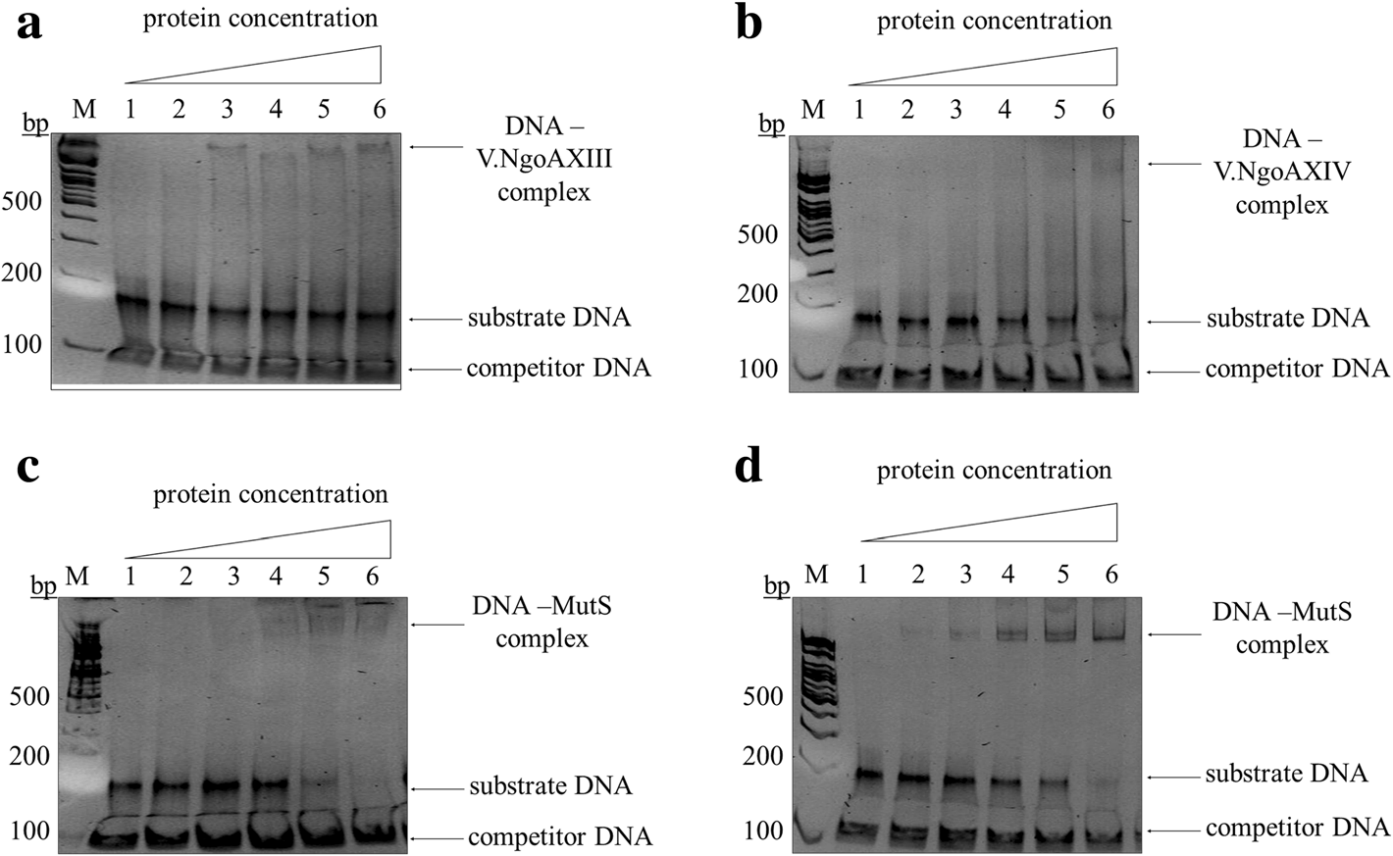
DNA binding by V.NgoAXIII, V.NgoAXIV endonucleases and MutS protein. DNA binding by (**a**) V.NgoAXIII endonuclease in the absence of 1 mM ATP; **b** V.NgoAXIV endonucleases in the absence of 1 mM ATP; **c** MutS protein in the absence of 1 mM ATP; **d** MutS protein in the presence of 1 mM ATP. The presented results were obtained using M.NgoA302P-sub (GTCGGT/ACCGGC) for V.NgoAXIII and M.NgoA1175P-sub (CTGG/CCGG) substrate DNA for V.NgoAXIV. Lanes are indicated as follows: M - GeneRuler™ DNA Ladder Mix (100, 200, 300, 400, 500, 600, 700, 800, 900, 1000, 1200, 1500, 2000, 2500, 3000, 3500, 4000, 5000, 6000, 8000 and 10,000 bp) (Thermo Scientific), 1–0 μM protein, 2–0.15 μM protein, 3–0.3 μM protein, 4–0.45 μM protein, 5–0.6 μM protein, 6–0.75 μM protein. The 94-bp competitor DNA was used in the experiments, which were performed in triplicate. Representative images are shown

In this analysis, 0.3 μM of V.NgoAXIII bound 10% of the substrate DNA in the presence of ATP, and a further increase in the amount of endonucleases did not increase the amount of bound DNA (Fig. 9a). Additionally, 0.75 μM of V.NgoAXIV (the second gonococcal Vsr endonuclease) bound 15% of the substrate DNA in the presence of ATP (Fig. 9b). The same results were obtained for the reaction carried out in the absence of ATP (data not shown).

In turn, 0.75 μM of MutS protein bound 90% of the substrate DNA. However, the DNA–MutS protein complexes were formed only in the presence of ATP (Fig. 9c and d).

In all cases, we did not observe binding of the competitor DNA by gonococcal Vsr endonucleases or MutS protein, indicating the specificity of the DNA binding by the studied proteins.

In conclusion, both gonococcal Vsr endonucleases and MutS protein bound the same mismatched sequences; however, the efficiency of DNA binding by MutS protein was dramatically increased compared with Vsr endonucleases, but only in presence of ATP.

## Discussion

DNA repair systems are crucial for all living organisms because they ensure genomic stability and integrity. The correct functioning of such systems relies on the interactions between proteins involved in DNA repair. In this work, for the first time, we demonstrated the mutator phenotype of gonococci with a disrupted *ngoAXIIIV* or *ngoAXIVV* gene, thereby indicating the in vivo engagement of V.NgoAXIII and V.NgoAXIV endonucleases in DNA repair. And by demonstration of the mutator phenotype of *N. gonorrhoeae mutL::km* and *N. gonorrhoeae mutS::km* mutants we confirmed the results of Criss et al. [16], who showed the involvement of MutL and MutS protein in DNA repair. However, for *E. coli* mutants with disrupted *ecoKDcmV*, *mutS* or *mutL* genes, strong mutator effects were noted [27, 28], and we have observed a relatively modest increase in the frequency level of spontaneous mutation for *ngoAXIIIV*, *ngoAXIVV*, *mutL* and *mutS* mutants of *N. gonorrhoeae*. An analogous level of the frequency of spontaneous mutation has also been noted for *mutL* and *mutS* mutants of *N*. *gonorrhoeae* by Criss et al. [16] and for *mutS* and *mutL* mutants of *N. meningitidis* (Additional file 1: Figure S3) [18, 29, 30]. Differences in mutation frequency between *N. gonorrhoeae* and *E. coli* with disrupted *vsr*, *mutL* or *mutS* genes may result from differences between the DNA repair pathways of these microorganisms. We also cannot rule out the possibility that in gonococcus, a few DNA repair pathways are engaged in the prevention of spontaneous mutations.

Furthermore, we investigated the interactions between the Vsr endonucleases, MutL and MutS proteins of *N. gonorrhoeae* FA1090. Thus, *N. gonorrhoeae* FA1090 is the first β-proteobacterium and the second of all microorganisms for which the physical and functional interactions between a Vsr endonuclease and MutL and MutS proteins have been demonstrated. As mentioned above, until now, the influence of MutL and MutS proteins on Vsr endonuclease activity has only been investigated for *E. coli* proteins. However, the results for *E. coli* proteins indicate a discrepancy concerning the involvement of the MutS protein in in vitro DNA cleavage. According to Monastiriakos et al. [3], MutL alone stimulates Vsr endonuclease activity, but as is demonstrated in [4], the activity of V.EcoKDcm endonuclease can be stimulated by MutL protein only when both MutS and ATP are also present in the reaction.

Here, we have shown that, although the gonococcal Vsr endonucleases digested DNA in the absence of any accessory proteins [12], the efficiency of in vitro DNA cleavage catalyzed by V.NgoAXIII and V.NgoAXIV was significantly increased in the presence of MutL_*Ngo*_ protein. This stimulatory effect was demonstrated for all tested DNA substrates; thus, we can conclude that the MutL_*Ngo*_ protein alone is sufficient to stimulate DNA cleavage by the gonococcal Vsr endonucleases, irrespective of the MutS protein and the nucleotide context of the T:G mismatch. As a consequence of the stimulation of V.NgoAXIII and V.NgoAXIV activity by MutL proteins, only a double molar excess of Vsr endonuclease over the substrate DNA was sufficient to completely digest the substrate DNA instead of the 10-fold excess required in the absence of MutL [12]. Therefore, MutL could allow for reduced expression and synthesis of Vsr endonucleases in cells. This finding is particularly important since *N. gonorrhoeae* FA1090 encodes two Vsr endonucleases and five active C5MTases that catalyze the creation of m5C, representing putative hot spots and a source of T:G mismatches. Lowering the amount of Vsr endonuclease required for complete digestion of the substrate DNA may allow efficient removal of T:G mismatches without the requirement for increased synthesis of Vsr.

Although both gonococcal Vsr endonucleases are stimulated by MutL proteins, there is a difference in the level of stimulation, which could result from the phylogenic distance of the V.NgoAXIII and V.NgoAXIV endonucleases, which belong to different groups [12]. V.NgoAXIV endonuclease, which belongs to the same group as the V.EcoKDcm endonuclease, is less susceptible to stimulation by MutL proteins. In turn, V.NgoAXIII endonucleases belonging to the second group of Vsr enzymes are more sensitive to the presence of MutL proteins. This difference is especially important because V.NgoAXIII recognizes the T:G mismatch in all nucleotide contexts, thus providing more comprehensive protection from the effects of m5C deamination; however, its lack of specificity may result in the need for more stringent regulation of its activity.

For the first time, we also demonstrated that the endonucleolytic activity of the Vsr endonucleases is stimulated not only by the MutL protein of the same organism, but also by that encoded by a distantly-related bacterial species. The capability of *E. coli* MutL protein to stimulate gonococcal Vsr endonucleases may result from the high similarity of amino acid sequences between *N*. *gonorrhoeae* and *E. coli* MutL proteins and the high evolutionary conservation of MutL proteins. Such conservation of the N-terminal domain has been observed for MutL proteins from different bacterial species [31] and may indicate a universal mechanism of Vsr activity regulation by MutL protein that involves its N-terminal domain. Indeed, in the *E. coli* MutL protein, the N-terminal domain is responsible for physical interactions with V.EcoKDcm endonuclease [4].

MutS protein also influences the efficiency of the reaction catalyzed by V.NgoAXIII and V.NgoAXIV. As we demonstrated in vitro, the presence of MutS hindered or completely inhibited DNA cleavage by gonococcal Vsr endonucleases. Such an effect was not observed for *E. coli* Vsr activity when the purified MutS protein was added in vitro to V.EcoKDcm, even at five-fold excess of MutS to V.EcoKDcm [4]. As we demonstrated, the inhibitory effect of the gonococcal MutS on the efficiency of the reaction catalyzed by Vsr endonucleases did not result from physical interactions between the endonucleases and the MutS protein, as these proteins did not interact with each other. Furthermore, analysis of the structure and spatial architecture of the domains responsible for binding T:G mismatches in the MutS and Vsr proteins indicates that simultaneous binding of mismatches by these proteins is impossible [5]. However, it may result from competition between Vsr endonuclease and MutS protein and from the higher affinity of MutS protein to the mismatch site than the V.NgoAXIII and V.NgoAXIV endonucleases. Thus, MutS can physically block the site for Vsr binding. The observed inhibitory effect of MutS on DNA digestion by Vsr is offset in the presence of MutL and ATP. The different levels of Vsr endonuclease activity exerted by gonococcal MutL and MutS proteins observed in vitro should be further investigated in vivo in a physiological context.

## Conclusions

The gonococcal Vsr endonucleases are engaged in DNA repair in vivo, and the activity of gonococcal endonucleases is influenced by the accessory proteins MutL and MutS. Thus, these proteins may be involved in the correct functioning of the VSP system by impacting Vsr endonuclease activity. Moreover, the influence of MutL protein of *E. coli* on the activity of the gonococcal Vsr endonucleases implies a universal mechanism regulation of DNA cleavage by the Vsr endonucleases.

## Methods

### Bacterial strains, plasmids, media, and growth conditions

*E. coli* ER1821 [F^−^*glnV44 e14*^*−*^*(McrA*^*−*^*) rfbDI? relA1? endA1 spoT1? thi*^*−*^*Δ(mcrC-mrr)114::IS10* (r^−^m^−^)] (New England Biolabs), BL21(DE3)pLys [*F*^*−*^*ompT gal dcm lon hsdS*_*B*_*(r*_*B*_^*−*^*m*_*B*_^*−*^*) λ(DE3)*] (Novagen), M15 [K12 Km^R^ (pREP4) Nal^S^ Str^S^ Rif^S^ Thi^−^ Lac^−^ Ara^+^ Gal^+^ Mtl^−^ F^−^ RecA^+^ Uvr^+^ Lon^+^] (Qiagen) and *E. coli* R721 [*supE thy* Δ(*lac*-*proAB*) F′ (*proAB*^+^*lacI*^*q*^*lac* ΔM15) *glpT*::O-P_434/P22_*lacZ*] [32] were used for gene cloning, protein expression and bacterial two-hybrid system experiments. These strains and their derivatives were grown in Luria-Bertani (LB) broth [33] supplemented when necessary with the following antibiotics: chloramphenicol (34 μg/ml), ampicillin (100 μg/ml) or kanamycin (25, 30 or 100 μg/ml).

The wild-type *N. gonorrhoeae* strain FA1090 and its derivatives were grown on plates of GC agar base (Difco) supplemented with 1% (v/v) hemoglobin and 1% (v/v) Kellogg’s supplement at 37 °C in 5% CO_2_ or in GC broth supplemented with 1% Kellogg’s supplement and 0.043% (v/v) NaHCO_3_ [20]. When required, media were supplemented with 30 μg/ml kanamycin and/or 0.75–2 μg/ml chloramphenicol.

Prior to each experiment, the piliation and Opa phenotypes of the *N. gonorrhoeae* strains employed were determined by microscopic examination of colony morphology. Only piliated gonococci were utilized in experiments and all strains exhibited the same piliation state.

### Construction of *N*. *gonorrhoeae* mutants and complemented strains

*N*. *gonorrhoeae* mutants and complemented strains were achieved by the gene replacement method [20]. To achieve this purpose, *N*. *gonorrhoeae* FA1090 cells were transformed with the plasmids described below.

All plasmids were prepared in *E. coli* ER1821 cells. First, the gonococcal *ngoAXIVV* (flanked by a sequence of ~ 500 bp), *ngoAXIIIV*, *mutL* and *mutS* genes were amplified from the chromosomal DNA of *N. gonorrhoeae* FA1090. The amplified DNA fragments were digested with appropriate restriction endonucleases and cloned into vector DNAs cleaved with the same enzymes (Additional file 1: Table S2). The *ngoAXIIIV* gene was cloned into pBluescript KS II (+), the *ngoAXIVV* gene into pUC19 and the *mutL* and *mutS* genes into the vector pMPMA4Ω [34]. This procedure generated the following plasmids: pBluescript KS II (+)::*ngoAXIIIV*, pUC19::*ngoAXIVV*, pMPMA4Ω::*mutL* and pMPMA4Ω::*mutS* (Additional file 1: Table S2).

Next, a kanamycin resistance (Km^R^) or chloramphenicol (Cm^R^) gene cassette was inserted into the cloned genes. The Km^R^ cassette was excised from plasmid pDIY-km [35] using either BamHI or SmaI. The Km^R^ cassette obtained by excision with BamHI was used for inactivation of the *ngoAXIIIV*, *mutL*, and *mutS* genes, while that with SmaI used to inactivate the *ngoAXIVV* gene. Thus, pBluescript KS II (+)::*ngoAXIIIV + km*, pUC19::*ngoAXIVV + km*, pMPMA4Ω::*mutL + km* and pMPMA4Ω::*mutS + km* were obtained. The Cm^R^ cassette was excised from pKRP10 plasmid [36] by SmaI and used to generate the pMPMA4Ω::*mutL + cm* and pMPMA4Ω::*mutS + cm* plasmid constructs (Additional file 1: Table S2). Where necessary, the cohesive termini of the DNA fragments used to construct the suicide vectors were modified by treatment with Klenow Fragment of DNA polymerase I or T4 DNA polymerase prior to ligation.

The obtained suicide plasmids were used to transform *N. gonorrhoeae* using the method reported by Dillard [20]. pBluescript KS II (+)::*ngoAXIIIV + km*, pUC19::*ngoAXIVV + km*, pMPMA4Ω::*mutL + km* or pMPMA4Ω::*mutS + km* was used for the transformation of *N. gonorrhoeae* wild-type, yielding *N. gonorrhoeae ngoAXIIIV::km*, *N. gonorrhoeae ngoAXIVV::km*, *N. gonorrhoeae mutL::km*, and *N. gonorrhoeae mutS::km* mutants*.* pMPMA4Ω::*mutL + cm* and pMPMA4Ω::*mutS + cm* plasmids were used to transform *N. gonorrhoeae ngoAXIIIV::km* or *N. gonorrhoeae ngoAXIVV::km*, allowing construction of the *N*. *gonorrhoeae ngoAXIIIV::km mutL::cm*, *N*. *gonorrhoeae ngoAXIVV::km mutL::cm*, *N*. *gonorrhoeae ngoAXIIIV::km mutS::cm*, and *N*. *gonorrhoeae ngoAXIVV::km mutS::cm* mutants.

Transformants were selected by their resistance to kanamycin or chloramphenicol, and the mutants and occurrence of double cross-over homologous recombinants were verified by PCR and Southern blot analysis. The presence of only one PCR product that corresponded in size to the sum of the mutated gene (414, 423, 1977 and 2595 bp for *ngoAXIIIV*, *ngoAXIVV*, *mutL* and *mutS* gene, respectively) and the Km^R^ cassette gene (950 bp) or Cm^R^ (842 bp) was treated as proof of successful mutagenesis. The amplified fragments were also fully sequenced to further verify the desired insertions. In addition, Southern blotting was carried out using non-radioactively labeled antibiotic resistance gene cassette (Ab^R^) probes (DIG-High Prime, Roche) to confirm the presence of single copy insertions.

For complementation of the *N*. *gonorrhoeae* mutants, wild-type copies of the appropriate genes were inserted into the chromosomes of the mutants in the intergenic region between the *ngo0275* and *ngo0274* genes [37]. This intergenic region plus ~ 500 bp flanking sequences was amplified from the *N. gonorrhoeae* FA1090 chromosome using Phusion High*-*Fidelity DNA Polymerase (Thermo Scientific) with primers iga and trpB (Additional file 1: Table S1). The PCR amplicon was digested with SalI and EcoRI, cloned into vector pMPMA4Ω cleaved with the same enzymes. The resulting construct, pMPMA4Ω::IgaTrpB, was then used as template in ExSite PCR with the primers exit1left and exit1right. Simultaneously, the Opa promoter was amplified from the chromosome of the *N. gonorrhoeae* FA1090 strain by PCR using Phusion High*-*Fidelity DNA Polymerase (Thermo Scientific) with primers opaleft and oparight (Additional file 1: Table S1). Both amplicons were digested with HindIII and Mph1103I and then ligated to generate the construct pMPMA4Ω::IgaTrpBOpa.

This plasmid was then digested with HindIII and ligated to a Cm^R^ cassette with HindIII cohesive termini excised from pKRP10 [36] to generate plasmid pMPMA4Ω::IgaTrpBOpaCm. This construct was used as template in ExSite PCR with primers smatrpB and Nheiga. The amplicon was then digested with SmaI and NheI and ligated to DNA fragments representing the *ngoAXIVV*, *ngoAXIIIV*, *mutL* or *mutS* genes amplified from the chromosome of *N. gonorrhoeae* FA1090 with the appropriate primers (Additional file 1: Table S1) and digested with the same restriction enzymes. These ligations produced constructs pMPMA4Ω::IgaTrpBOpaCmVNgoAXIIIV, pMPMA4Ω::IgaTrpBOpaCmVNgoAXIV, pMPMA4Ω::IgaTrpBOpaCmMutL and pMPMA4Ω::IgaTrpBOpaCmMutS, in which the respective gonococcal genes were fused to the constitutive Opa promoter.

These plasmids were linearized by digestion with EcoRV (*ngoAXIIIV*, *mutL* or *mutS* gene constructs) or BamHI (*ngoAXIVV* gene construct) and used to transform piliated *N. gonorrhoeae* mutant strains according to the method reported by Dillard [20]. In each case, the plasmid used to transform the mutant strain carried the gene required to complement that particular mutation. Transformants isolated by selection with kanamycin and chloramphenicol were then examined by PCR. *In cis* complementation of the *N*. *gonorrhoeae* mutants was confirmed by the presence of two PCR products: one large, corresponding in size to the sum of the *ngoAXIIIV*, *ngoAXIVV*, *mutL* or *mutS* gene and the Ab^R^ cassette, and the other small, corresponding in size to the wild-type gene alone. The same primers that were used for amplification of particular genes were applied (Additional file 1: Table S1). Insertion of a functional copy of the gene of interest into the gonococcal chromosome between the *ngo0275* and *ngo0274* genes was also verified by PCR using primers iga and trpB. A 1134-bp fragment was amplified from the untransformed *N. gonorrhoeae* mutant strains, but following complementation, the size of the amplicon was increased by the combined size of the Cm^R^ cassette (842 bp), Opa promoter (243 bp) and the gene of interest, i.e., 414, 423, 1977 and 2595 bp for the *ngoAXIIIV*, *ngoAXIVV*, *mutL* and *mutS* gene, respectively.

### Determination of the spontaneous mutation frequency and nature of the mutations

*N. gonorrhoeae* wild-type, mutant and complemented mutant strains were cultivated on GC agar for 16 h. The cells were then harvested from the plates, suspended in GC broth (10^9^ cells/ml of each strain) and 0.1 ml aliquots of each strain were plated on GC agar base supplemented with 0.12 μg/ml rifampicin or 1 μg/ml nalidixic acid. In parallel, the gonococcal cells were diluted 10^− 6^, and 0.1 ml aliquots were plated on GC agar base without antibiotics to determine the total viable cell number. All plates were incubated at 37 °C in 5% CO_2_ for 48 h, and then the colony numbers were counted. The proportion of rifampicin-resistant cells was determined for the wild-type strain and gonococcal mutants to assess whether disruption of the *ngoAXIIIV* or *ngoAXIVV* genes resulted in an increase in the spontaneous mutation frequency. The mean frequency of spontaneous mutations was determined from 6 independent experiments. The significance of any differences was calculated using the two-tailed heteroscedastic Student’s *t*-test (*p* < 0.05).

To determine the nature of the mutations, gonococcal wild-type and mutants were cultivated for determination of the spontaneous mutation frequency and plated on GC agar supplemented with 0.12 μg/ml rifampicin. Next, the central conserved region of the *rpoB* gene [21–23] was PCR-amplified from rifampicin-resistant colonies using Phusion High*-*Fidelity DNA Polymerase. The amplified fragments were sequenced using primers rpoBNGF and rpoBNGR, and the nucleotide sequences were compared to the “wild-type” *rpoB* gene sequence. Thirty rifampicin-resistant colonies of each *N. gonorrhoeae* strain were analyzed in this manner.

### Bacterial two-hybrid system

The bacterial two-hybrid system is based on the repression of β-galactosidase activity, as developed and described by Di Lallo et al. [32]. In this system, a chimeric operator is recognized by a hybrid repressor formed by two chimeric monomers, one domain of which is composed of heterologous proteins. Only if these proteins efficiently dimerize in vivo is the functional repressor formed, binding the chimeric operator and terminating the synthesis of a downstream *lacZ* gene.

First, *mutL*_*Ngo*_, *mutS*, *ngoAXIIIV* or *ngoAXIVV* genes were amplified by PCR using the chromosomal DNA of *N. gonorrhoeae* FA1090 as template. Primer names and sequences are listed in Additional file 1: Table S1. These amplicons were cloned into the SalI–BamHI sites of vectors pC434 and pC22 [32] using a standard cloning procedure. Genes *ngoAXIIIV* and *ngoAXIVV* were cloned into pC22, *mutL*_*Ngo*_ into pC434, and *mutS* into the pC434 and pC22 vectors.

Subsequently, *E. coli* R721 cells were transformed with pC434- and pC22-derived plasmids (Additional file 1: Table S2). In this strain, there was a chimeric operator upstream of the *lacZ* gene encoding β-galactosidase, formed by two hemi-sites of P22 and a 434 phage operator. This operator was recognized and bound only by a hybrid repressor, consisting of two chimeric monomers. One of them contained the N-terminal domain of the repressor of phage 434, and the other contained that of phage P22. These domains were fused with the sequence of the heterologous proteins, the interaction abilities of which were investigated. If the studied proteins physically interacted with one another, a functional chimeric repressor was formed, and the N-terminal part of the hybrid repressor bound to the sequence of the chimeric operator, leading to the repression of β-galactosidase synthesis. If the tested proteins did not interact with one another, a functional repressor was not formed and the β-galactosidase gene was expressed.

To examine the interactions between gonococcal Vsr endonucleases, MutL and MutS proteins, the following combinations of two plasmids (Additional file 1: Table S2) were used for co-transformation: (i) pAK24 and pAK26 to investigate the interactions between V.NgoAXIII and MutL_*Ngo*_, (ii) pAK25 and pAK26 to study the interactions between V.NgoAXIV and MutL_*Ngo*_, (iii) pAK24 and pAK28 to investigate the interactions between V.NgoAXIII and MutS, (iv) pAK25 and pAK28 to study the interactions between V.NgoAXIV and MutS, and (v) pAK26 and pAK27 to analyze the interactions between MutL_*Ngo*_ and MutS proteins.

Next, six colonies of fresh transformants were used for inoculation of 10 ml LB supplemented with 0.1 mM IPTG and the appropriate antibiotics, and grown at 37 °C until the optical density at 600 nm (OD_600_) of the culture reached 0.3–0.4. Subsequently, a β-galactosidase assay was performed to analyze protein–protein interactions, and the ratio of β-galactosidase activity for the *E. coli* R721 strain with both plasmids carrying genes for the two investigated proteins to that of the plasmid-less R721 strain was estimated. β-galactosidase activity (given in Miller Units) was calculated according to the following equation: Miller units of β-galactosidase activity = (1000 × (A_420−_ 1.75×A_550_))/T×V×A_600_, where A is the absorbance at 420, 550 or 600 nm, V – the volume of the sample [ml]; T – reaction time in minutes.

Each set of experiments contained both negative and positive controls. *E. coli* R721 cells transformed with only one plasmid (pC22-or pC434-derived) were used as the negative control, while *E. coli* R721 harboring plasmids pcI_434_22 and pcIP_22_434 (containing the repressor of phage 22 and 434, respectively) was used as the positive control. All experiments were repeated at least six times, and their results were averaged. The significance of any differences from six independent experiments was calculated using the two-tailed heteroscedastic Student’s *t*-test (*p* < 0.05).

### Gene cloning and protein expression and purification

Cloning of the *ngoAXIIIV* and *ngoAXIVV* genes into pET28a(+) (Novagen) and pQE-30 (Qiagen) vectors, respectively, has been previously described in [12], along with the protocol for expression and purification of the V.NgoAXIII and V.NgoAXIV endonucleases by metal affinity chromatography.

*mutL*_*Ngo*_ (acc. no. AAW89461.1) (1977 bp) and *mutS* (acc. no. AAW90543) (2595 bp) genes were amplified by PCR from chromosomal DNA of *N. gonorrhoeae* FA1090 (acc. no. NC_002946) using primers MutLfor/MutLNgoR and MutSNheI/MutSNgoR, respectively (Additional file 1: Table S1). The *mutL*_*Ngo*_ gene was cloned into the NheI – HindIII sites of pET28a(+), resulting in the formation of plasmid pAK20, and the *mutS* gene was cloned into the NheI–HindIII sites of pET28a(+) to yield plasmid pAK21 (Additional file 1: Table S2).

*mutL*_*E.coli*_ (1848 bp) (acc. no. GU134327) gene was amplified by PCR from chromosomal DNA of *E. coli* K-12 substr*.* MG1655 (acc. no. NC_000913.3) using primers MutLEcoFor and MutLEcoRev (Additional file 1: Table S1) and cloned into the NheI–XhoI sites of pET28a(+), resulting in plasmid pAK22 (Additional file 1: Table S2).

The genes for MutL_*Ngo*_, MutL_*E.coli*_ and MutS proteins were cloned in such a way that the resulting proteins contained a vector-encoded amino terminal His-Tag.

The *mutL*_*Ngo*_*, mutS* and *mutL*_*E.coli*_ genes were expressed by autoinduction [38]. For this purpose, a single bacterial colony, generated by fresh transformation of *E. coli* BL21(DE3)pLys (pAK20), *E. coli* BL21(DE3)pLys (pAK21) or *E. coli* BL21(DE3)pLys (pAK22), was used to inoculate 10 ml of ZYP medium [38] supplemented with 1 mM MgSO_4_, 0.8% (v/v) glucose, 1×NPS (0.5 M (NH_4_)_2_SO_4_, 1 M KH_2_PO_4_, 1 M Na_2_HPO_4_) and kanamycin (100 μg/ml). Cultures were incubated at 37 °C for 5 h. Subsequently, 1.75 ml of the bacterial cultures was transferred to 320 ml of ZYP medium supplemented with 1 mM MgSO_4_, 0.5% (v/v) glycerol, 1×NPS, 0.05% (v/v) glucose, 0.2% (v/v) lactose, and kanamycin (100 μg/ml). Incubation at 37 °C was continued for an additional 16 h. The culture was centrifuged, and the obtained bacterial pellet was resuspended in 10 ml of a sonication buffer containing 50 mM NaHPO_4_ pH 8.0, 500 mM NaCl, 10 mM imidazole, 10 mM 2-mercaptoethanol, 0.1% (v/v) Tween 20, 55 μM PMSF and 1 tablet of Complete Mini (an EDTA-free protease inhibitor cocktail, Roche Diagnostics). After sonication, the cellular debris was removed by centrifugation at 40000 *g* for 1 h, and the supernatant was applied to a 0.5-ml Ni-NTA Agarose column that had been previously equilibrated with 100 ml of the sonication buffer. The column was then washed with 100 ml of a washing buffer containing 50 mM NaHPO_4_ pH 8.0, 500 mM NaCl, 25 mM imidazole and 10% (v/v) glycerol. The proteins were eluted with increasing concentrations of imidazole (from 50 mM to 500 mM) in the same buffer. MutL_*Ngo*_, MutL_*E.coli*_ and MutS proteins were eluted with 200–250 mM imidazole. The homogeneity of the purified enzymes was assessed by 10% or 12% SDS PAGE (Additional file 1: Figure S4). Protein concentrations were determined using Bradford Reagent (Sigma-Aldrich) with bovine serum albumin (BSA) as a protein standard. The total yield of the purified protein was ~ 3 mg for MutL_*Ngo*_, ~ 2.5 mg for MutL_*E.coli*_, ~ 2 mg for MutS, and ~ 1.5 mg for each of the V.NgoAXIII and V.NgoAXIV endonucleases.

### Protein affinity chromatography

Protein affinity chromatography was carried out analogously to [39–41] with slight modifications. To achieve this purpose, purified V.NgoAXIII (250 μg), V.NgoAXIV (250 μg) or MutL_*Ngo*_ (250 μg) proteins were coupled to Affi-Gel^®^ 10 (ester activated agarose) according to the supplier (Bio-Rad) in 0.1 M NaHCO_3_, pH 8.9 (V.NgoAXIII or V.NgoAXIV) or 0.1 M MOPS pH 7.5, 50 mM CaCl_2_ (MutL_*Ngo*_). Then, residual active groups of the resin were blocked by incubating the gel with 1 M ethanolamine, pH 8.9. After transfer to a chromatography column, excess soluble protein was removed by washing the gel with buffer A (25 mM Tris pH 7.5, 1 M NaCl), and the column was then equilibrated with buffer B (25 mM Tris pH 7.5, 50 mM NaCl). Then: (i) MutL_*Ngo*_ protein (250 μg in buffer B with 2 mM ATP and 5 mM MgCl_2_) was applied to the V.NgoAXIII, V.NgoAXIV or control columns; (ii) MutS protein (250 μg in buffer B with 2 mM ATP and 5 mM MgCl_2_) was applied to MutL_*Ngo*_, V.NgoAXIII, V.NgoAXIV or control columns, which were subsequently washed with buffer B. Proteins were eluted with buffer C (25 mM Tris pH 7.5, 250 mM NaCl). Proteins were separated by electrophoresis on SDS-polyacrylamide gels and blotted onto an Immun-Blot™ PVDF membrane (Bio-Rad). Since the proteins contained a His-Tag, Western blot analysis was carried out using a 6×-His Tag Monoclonal Antibody (ThermoFisher) and Goat Anti-Mouse IgG, Alkaline Phosphatase conjugated (ThermoFisher) antibodies according to the manufacturer’s recommendations. As a control resin, BSA-coupled gel was used.

### Preparation of substrate DNA for Vsr activity assessment

Preparation of substrate DNA for Vsr endonuclease activity and specificity study was performed as described and presented in [12, 13]. Construction of DNA substrates containing two T:G mismatches and the principle of an assay to demonstrate the activity of Vsr endonuclease are presented on Additional file 1: Figure S5. Briefly, two oligonucleotides (Sigma-Aldrich), 5′ATATTCAAACTGGCGCCGAGCGTATGCCGCATGACCTTTCCCATCTTGGCTTCCTTGCTGGTCAGATTGGTCGTCTTATTACCATTTCAACTACTNNNNNNGATATCNNNNNNCGACTCC 3′ (120-mer) and 5′AGCAAGGCCACGACGCAATGGAGAAAGACGGAGAGCGCCAACGGCGTCCATCTCGAAGGAGTCGNNNNNNGATATCNNNNNNAGTAGTTG 3′ (90-mer), were used to prepare the substrate DNA. The DNA sequence of these oligonucleotides was a derivative of the M13 bacteriophage DNA. Each oligonucleotide contained a 33 nt-long complementary region (underlined). NNNNNN indicates the sequence recognized by the studied Vsr endonucleases. Additionally, the sequence in which thymine is mismatched to guanine is indicated in italics. These oligonucleotides were mixed and hybridized at 95 °C for 5 min in 1×SSC (15 mM sodium citrate pH 7.2, 150 mM NaCl) and then slowly cooled to room temperature. Subsequently, single-stranded ends were filled using Polymerase I Klenow Fragment (Thermo Scientific) in the presence of dNTPs, under the conditions recommended by the manufacturer. Each substrate DNA contained two copies of the same sequence carrying a T:G mismatch (Additional file 1: Table S3). Additionally, substrate DNA containing a T:T mismatch was created. The control substrate DNAs did not contain any mismatches, and the AGCGCC sequence was substituted for the region indicted by NNNNNN. All substrates used are listed in Additional file 1: Table S3.

### Analysis of the activity of Vsr endonucleases in the presence of MutL and MutS proteins

The in vitro endonucleolytic activity of Vsr endonucleases (V.NgoAXIII, V.NgoAXIV) in the presence of MutL_*Ngo*_ or MutL_*E.coli*_ and MutS proteins was assayed by incubation of 0.15 μM substrate DNA with 0.3 μM of a Vsr endonuclease, in a final volume of 20 μl containing either 10 mM Tris-HCl (pH 7.5), 10 mM MgCl_2_ and 0.1 mg/ml BSA (for V.NgoAXIII), or 66 mM Tris-acetate (pH 7.9 at 37 °C), 20 mM magnesium acetate, 132 mM potassium acetate and 0.2 mg/ml BSA (for V.NgoAXIV), at 37 °C for 60 min. The effect of MutL and MutS proteins on the activity of Vsr endonucleases was studied using 0.3–0.6 μM of the purified MutL and MutS proteins. Additionally, when needed, 1 mM ATP was added. Control reactions were carried out with (i) a Vsr endonuclease and BSA, with or without 1 mM ATP; (ii) BSA only, with or without 1 mM ATP; or (iii) a MutL_*Ngo*_ protein with or without 1 mM ATP. The cleavage products were analyzed using 10% neutral polyacrylamide gels, and DNA was visualized by ethidium bromide staining. The appearance of two product bands (~ 110 and ~ 80 bp) after treatment of the substrate DNA (~ 190 bp) with Vsr endonuclease supported its hydrolytic activity. The amount of DNA in substrate and product bands was determined using a Gel/ChemiDoc (Bio-Rad) analyzer, quantified with the accompanying software package (Quantity One) (Bio-Rad), and expressed as a fraction of the total intensity of DNA as visualized by ethidium bromide staining. Then, the electrophoresis profiles were used to quantify the efficiency of cleavage of the substrate DNA by Vsr endonucleases. The efficiency of the reaction was calculated based on analysis of the rate constants determined by fitting the data to a first-order rate equation: % product_*t*_ = 100[1-exp(−*k*_*st*_)] (where *t* is the time and *k*_st_ is the first-order rate constant) [3, 4, 12, 42, 43] using Origin 6.1 software (OriginLab, USA). The significance of any differences from five independent experiments was calculated using the two-tailed heteroscedastic Student’s *t*-test (*p* < 0.05).

Additionally, to demonstrate the nicking activity and specificity of the gonococcal Vsr endonucleases, the reaction products were also separated in a denaturing gel as was used for the characterization of Vsr endonuclease encoded by *E. coli* [10, 44, 45] (Additional file 1: Figure S6).

### DNA binding by gonococcal V.NgoAXIII and V.NgoAXIV endonucleases and MutS protein

Protein–DNA complexes were detected using a gel electrophoresis mobility shift assay (EMSA). Binding of DNA by the investigated proteins was studied by incubation of 0.15 μM substrate DNA with 0.15–0.75 μM protein in a final volume of 20 μl, which contained 10 mM Tris-HCl (pH 7.5), 10 mM CaCl_2_, 0.1 mg/ml BSA and a nonspecific competitor DNA (0.032 μM of DNA without any mismatches). The competitor DNA was obtained by PCR amplification using chromosomal DNA of *N. gonorrhoeae* FA1090 as template, primers 16S RT F and 16S RT R, and PfuUltra DNA Polymerase (Stratagene). Additionally, when needed, 1 mM ATP was added. Next, 4 μl of 5% sucrose was added to the reaction mixture before loading the DNA–protein complexes in a gel. The complexes were separated on neutral 5% (w*/*v) polyacrylamide (19:1 acrylamide:bisacrylamide) gels supplemented with 1 mM MgCl_2_, 0.5 mM DTT, cast in 22.5 mM Tris base, 22.5 mM boric acid, 0.5 mM EDTA (pH 8.0) and run in 22.5 mM Tris base, 22.5 mM boric acid, 0.5 mM EDTA (pH 8.0). DNA–protein complexes were visualized by ethidium bromide staining. All experiments were repeated at least three times, and the results were averaged.

### Standard molecular biology procedures

The primers for DNA amplification were synthesized at the Institute of Biochemistry and Biophysics, Poland. The PCR reactions were carried out using Pfu DNA Polymerase (Thermo Scientific) or PfuUltra DNA Polymerase (Stratagene), according to the manufacturer’s recommendations.

All standard methods were carried out in accordance with the protocols described in [33].

### In silico analysis

DNA and protein sequences were compared with GenBank and SWISS-PROT databases on the BLAST server hosted by the National Center for Biotechnology Information (www.ncbi.nlm.nih.gov/blast).

### Enzymes and chemicals

Restriction enzymes, T4 DNA ligase, IPTG (isopropyl-β-D-thiogalactopyranoside), as well as DNA and protein markers were purchased from Thermo Scientific and used under conditions recommended by the manufacturers. Kits for DNA clean-up and plasmid DNA preparation were purchased from A&A Biotechnology, Poland. Ni-NTA Agarose was purchased from Qiagen. All other chemicals were purchased from Sigma-Aldrich, unless otherwise noted.

## Additional file

Additional file 1: **Table S1.** Primers used in the work. **Table S2.** Plasmids used in the work. **Table S3.** DNA substrates used in the work for the study the activity of gonococcal Vsr endonucleases. **Table S4.** The mutations occurred in the *rpoB* gene in the gonococcal mutants with disrupted *vsr* genes. **Table S5.** The activity of gonococcal Vsr endonucleases in the presence of the gonococcal MutL protein. The *k*_st_ values for all DNA substrates recognized by Vsr endonucleases that were used for plotting the graphs in Fig. 4C. **Table S6.** The activity of gonococcal Vsr endonucleases in the presence of MutL protein of *E. coli*. The *k*_st_ values for all DNA substrates recognized by Vsr endonucleases that were used to plot the graphs in Fig. 6. **Table S7.** The activity of gonococcal Vsr endonucleases in the presence of the gonococcal MutS protein. The *k*_st_ values for all DNA substrates recognized by Vsr endonucleases that were used to plot the graphs in Fig. 7C. **Figure S1.** Spontaneous mutation frequency in *N*. *gonorrhoeae* mutants with disrupted *ngoAXIIIV*, *ngoAXIVV*, *mutL*_*Ngo*_ or *mutS* genes assayed by nalidixic acid resistance. **Figure S2.** The presence of the MutL_*Ngo*_ protein decreases amount of Vsr endonuclease required to complete DNA digestion. The results for DNA substrates that are not presented in Fig. 5. **Figure S3.** Comparison of the increase in mutation frequency in different bacterial species with disrupted *mutL*, *mutS* or *vsr* genes. **Figure S4.** Purification of the V.NgoAXIII endonuclease, V.NgoAXIV endonuclease, MutL_*Ngo*_and MutS protein of *N. gonorrhoeae* FA1090 and the MutL protein of *E. coli.* **Figure S5.** Construction of DNA substrates containing two T:G mismatches and principle of an assay to demonstrate the activity of Vsr endonuclease. **Figure S6.** Construction of DNA substrates containing one T:G mismatch and principle of an assay to demonstrate the activity of Vsr endonuclease. (PDF 2718 kb)

## Abbreviations

C5MTase: C^5^-methyltransferase m5C 5-methylcytosine MutL_*E.coli*_ MutL protein of *E. coli* K-12 MutL_*Ngo*_ MutL protein of *N*. *gonorrhoeae* FA1090 VSP Very Short Patch repair system
