# Supplementary material for: Activity of Vsr endonucleases encoded by Neisseria gonorrhoeae FA1090 is influenced by MutL and MutS proteins
Source: BMC Microbiol. 2018 Aug 30;18:95. doi: 10.1186/s12866-018-1243-3 (PMC6116569; doi:10.1186/s12866-018-1243-3)
Supplement: Supplementary file 1 — Table S1. Primers used in the work. Table S2. Plasmids used in the work. Table S3. DNA substrates used in the work for the study the activity of gonococcal Vsr endonucleases. Table S4. The mutations occurred in the rpoB gene in the gonococcal mutants with disrupted vsr genes. Table S5. The activity of gonococcal Vsr endonucleases in the presence of the gonococcal MutL protein. The kst values for all DNA substrates recognized by Vsr endonucleases that were used for plotting the graphs in Fig. 4C. Table S6. The activity of gonococcal Vsr endonucleases in the presence of MutL protein of E. coli. The kst values for all DNA substrates recognized by Vsr endonucleases that were used to plot the graphs in Fig. 6. Table S7. The activity of gonococcal Vsr endonucleases in the presence of the gonococcal MutS protein. The kst values for all DNA substrates recognized by Vsr endonucleases that were used to plot the graphs in Fig. 7C. Figure S1. Spontaneous mutation frequency in N. gonorrhoeae mutants with disrupted ngoAXIIIV, ngoAXIVV, mutLNgo or mutS genes assayed by nalidixic acid resistance. Figure S2. The presence of the MutLNgo protein decreases amount of Vsr endonuclease required to complete DNA digestion. The results for DNA substrates that are not presented in Fig. 5. Figure S3. Comparison of the increase in mutation frequency in different bacterial species with disrupted mutL, mutS or vsr genes. Figure S4. Purification of the V.NgoAXIII endonuclease, V.NgoAXIV endonuclease, MutLNgoand MutS protein of N. gonorrhoeae FA1090 and the MutL protein of E. coli. Figure S5. Construction of DNA substrates containing two T:G mismatches and principle of an assay to demonstrate the activity of Vsr endonuclease. Figure S6. Construction of DNA substrates containing one T:G mismatch and principle of an assay to demonstrate the activity of Vsr endonuclease. (PDF 2718 kb) [file 12866_2018_1243_MOESM1_ESM.pdf]

Table S1. Primers used in the work.

Gene	Primer names	Primer sequences (5' → 3')[a]
ngoAXIIIV[b]	VsrASalF<br>VsrABamR	ACGCGTCGACCATGGATAAATTAACC<br>CGGGATCCTCATCTTGTTTGGATGA
ngoAXIIIV[d]	AK 300<br>AK 400	AGTTGGATCCGATAAATTAACCCCGGAG<br>GCATCAAGCTTTCATCTTGTTTGGATGATTTC
ngoAXIIIV[h]	VsrANheF<br>VsrAKomR	CTAGCTAGCATGGATAAATTAACCCC<br>CGCCCGGGTCATCTTGTTTGGATGATTTCTTTGAC
ngoAXIVV[b]	VsrBSalF<br>VSRBPRNEW	ACGCGTCGACCATGACCGATATTTTCAC<br>ATAGCATGGATCCTCAGACGGCATCTTTTATTTCCTC
ngoAXIVV[ef]	dlVsrBf<br>dlVsrB	CGCGGATCCTCCTTGGGGAAATCGTCTGAG<br>CCCAAGCTTGCCGCGCGTATGAAATCGTCATC
ngoAXIVV[h]	VsrBNheF<br>VsrBKomR	CTAGCTAGCATGACCGATATTTTCACTCCATCC<br>CGCCCGGGTCAGACGGCATCTTTTATTTTC
mutL$_{Ngo}$[b]	MutLSalF<br>MutLBamR	ACGCGTCGACCATGCCACGCATTGC<br>CGCGGATCCTTACTGTCCGCGCAAG
mutL$_{Ngo}$[c]	MutLfor<br>MutLNgoR	CTAGCTAGCATGCCACGCATTGCCG<br>GCAGAAGCTTTTACTGTCCGCGCAAGAACAGTGTGT

mutL_{Ngo}[g]	MutLNcoI<br>MutLNgoR	CATGCCATGGATGCCACGCATTGCCGC<br>GCAGAAGCTTTTACTGTCCGCGCAAGAACAGTGTGT
mutL_{Ngo}[h]	MutLNheF<br>MutLKomR	CTAGCTAGCATGCCACGCATTGCCGCCC<br>CGCCCGGGTTACTGTCCGCGCAAGAAC
mutL_{Ecoli}[c]	MutLEcoFor<br>MutLEcoRev	CTAGCTAGCATGCCAATTCAGGTCTTACCGCC<br>CCGCTCGAGTCACTCATCTTTCAGGGCTTTTATC
mutS[b]	MutSSalF<br>MutSBamR	ACGCGTCGACCATGTCCAAATCCGCC<br>CGGGATCCTTAAGATACGGATTTGCAC
mutS[c]	MutSNheI<br>MutSNgoR	ATAGCTAGCTCCAAATCCGCCGTTTCCCC<br>GGCAAGCTTTTAAGATACGGATTTGCACAAATCTTT
mutS[g]	MutSXbaI<br>MutSNgoR	GCTCTAGAATGTCCAAATCCGCCG<br>GGCAAGCTTTTAAGATACGGATTTGCACAAATCTTT
mutS[h]	MutSNheF<br>MutSKomR	CTAGCTAGCATGTCCAAATCCGCCGTTTCCCC<br>GCCCCGGGTTAAGATACGGATTTGCACAAATCTTTCAG
16S rRNA	16S RT F<br>16S RT R	GCGGGGTAGCAAACAGGAT<br>CGCGAGCTACGCTACCAAG
intergenic region	iga<br>trpB	ACGCGTCGACTATGTGGCCGGCGATATTG<br>CCGGAATTCAAAGCGCAGATGCAGGAAGC

between *ngo0275* and *ngo0274*[e]		
-	exit1left<br>exit1rigth	CCCGAAGCTTGAGTTCAGACGGC<br>CCAATGCATGCTTCGGACGGCATTTTTATCCAG
opa promoter	opaleft<br>oparigth	CCCAAGCTTGGGCGGATTATATCGGGTTC<br>CCAATGCATGGAATGACGGCGGAAAGATG
-	Nheiga<br>smatrpB	CTAGCTAGCGTCCGAAGCATGCATGGAATGAC<br>TCCCCCGGGGACTAACAAAGCTACAGCCTCAATGC
rpoB	rpoBNGF<br>rpoBNGR	GCCGATTTGATTGATTCGGAAACC<br>GCTTCGTCGTCATGGACACGGA

[a] The sequence recognized by restriction enzyme used to digestion of PCR product and cloning is underlined.

[b] Primers used for cloning into the pC434 or pC22 vectors.

[c] Primers used for cloning into the pET28a(+).

[d] Primers used for cloning into pBluescript KS II (+) vector.

[e] The intergenic region or *ngoAXIVV* gene were cloned with ~ 500-bp flanking sequences.

[f] Primers used for cloning into pUC19 vector.

[g] Primers used for cloning into pMPMA4Ω vector.

[h] Primers used for cloning into the pMPMA4Ω::IgaTrpBOpaCm plasmid.

Table S2. Plasmids used in this work.

Plasmid name	Gene	Description
pAK15	*ngoAXIIIV*	contains *ngoAXIIIV* gene encoding the V.NgoAXIII endonuclease cloned into the pET28a(+) vector [1].
pAK16	*ngoAXIVV*	contains *ngoAXIVV* gene encoding endonuclease V.NgoAXIV cloned into pQE-30 vector [1].
pAK20	*mutL*$_{Ngo}$	contains *mutL*$_{Ngo}$ gene encoding *N. gonorrhoeae* MutL$_{Ngo}$ protein cloned into pET28a(+) vector
pAK21	*mutS*	contains *mutS* gene encoding *N. gonorrhoeae* MutS protein cloned into pET28a(+) vector
pAK22	*mutL*$_{E.coli}$	contains *mutL*$_{E.coli}$ gene encoding *E. coli* MutL$_{E.coli}$ protein cloned into pET28a(+) vector
pAK24	*ngoAXIIIV*	contains *ngoAXIIIV* gene encoding

		V.NgoAXIII endonuclease cloned into pC22 vector
pAK25	*ngoAXIVV*	contains *ngoAXIVV* gene encoding V.NgoAXIV endonuclease cloned into pC22 vector
pAK26	$mutL_{Ngo}$	contains $mutL_{Ngo}$ gene encoding *N. gonorrhoeae* $MutL_{Ngo}$ cloned into pC434 vector
pAK27	*mutS*	contains *mutS* gene encoding *N. gonorrhoeae* MutS cloned into pC22 vector
pAK28	*mutS*	contains *mutS* gene encoding *N. gonorrhoeae* MutS cloned into pC434 vector
pBluescript KS II (+)::*ngoAXIIIV*	*ngoAXIIIV*	contains *ngoAXIIIV* gene encoding V.NgoAXIII endonuclease cloned into pBluescript KS II(+) vector
pBluescript KS II (+)::*ngoAXIIIV+km*	*ngoAXIIIV*	contains *ngoAXIIIV* gene encoding V.NgoAXIII endonuclease disrupted by a kanamycin resistance cassette
pUC19::*ngoAXIVV*	*ngoAXIVV*	contains *ngoAXIVV* gene encoding V.NgoAXIV endonuclease cloned

		into pUC19 vector
pUC19::*ngoAXIVV+km*	*ngoAXIVV*	contains *ngoAXIVV* gene encoding V.NgoAXIV endonuclease disrupted by a kanamycin resistance cassette
pMPMA4Ω::*mutL$_{Ngo}$*	*mutL$_{Ngo}$*	contains *mutL$_{Ngo}$* gene encoding MutL$_{Ngo}$ protein cloned into pMPMA4Ω
pMPMA4Ω::*mutL$_{Ngo}$+km*	*mutL$_{Ngo}$*	contains *mutL$_{Ngo}$* gene encoding MutL$_{Ngo}$ disrupted by a kanamycin resistance cassette
pMPMA4Ω::*mutS*	*mutS*	contains *mutS* gene encoding MutS protein cloned into pMPMA4Ω
pMPMA4Ω::*mutS+km*	*mutS*	contains *mutS* gene encoding MutS disrupted by a kanamycin resistance cassette
pMPMA4Ω::IgaTrpB	-	contains intergenic region between *ngo0275* and *ngo0274* cloned into pMPMA4Ω
pMPMA4Ω::IgaTrpBOpa	-	contains intergenic region between *ngo0275* and *ngo0274* with opa promoter

pMPMA4Ω::IgaTrpBOpaCm	-	contains intergenic region between *ngo0275* and *ngo0274*, opa promoter, and a chloramphenicol resistance cassette
pMPMA4Ω::IgaTrpBOpaCmVNgoAXIII	*ngoAXIIIV*	contains *ngoAXIIIV* gene encoding V.NgoAXIV endonuclease cloned into pMPMA4Ω::IgaTrpBOpaCm plasmid
pMPMA4Ω::IgaTrpBOpaCmVNgoAXIV	*ngoAXIVV*	contains *ngoAXIVV* gene encoding V.NgoAXIV endonuclease cloned into pMPMA4Ω::IgaTrpBOpaCm plasmid
pMPMA4Ω::IgaTrpBOpaCmMutL	$mutL_{Ngo}$	contains $mutL_{Ngo}$ gene encoding MutL_{Ngo} protein cloned into pMPMA4Ω::IgaTrpBOpaCm plasmid
pMPMA4Ω::IgaTrpBOpaCmMutS	*mutS*	contains *mutS* gene encoding MutS protein cloned into pMPMA4Ω::IgaTrpBOpaCm plasmid

Table S3. DNA substrates used in the work.

Substrate name	Sequence [a]
Control	5’ CAACTACT**AGCGCC**GATATC**AGCGCC**CGACTCC 3’<br>3’ GTTGATGA**TCGCGG**CTATAG**TCGCGG**GCTGAGG 5’
M.NgoAI-sub[b]	5’ CAACTACT**AGCGCT**GATATC**AG_T_GCT**CGACTCC 3’<br>3’ GTTGATGA**TCG_T_GA**CTATAG**TCGCGA**GCTGAGG 5’
M.NgoAII-sub[b]	5’ CAACTACT**GGCC**GATATC**GG_T_C**CGACTCC 3’<br>3’ GTTGATGA**C_T_GG**CTATAG**CCGG**GCTGAGG 5
M.NgoAIII”first”-sub	5’ CAACTACT**CCGCGG**GATATC**_T_CGCGG**CGACTCC 3’<br>3’ GTTGATGA**GGCGC_T_**CTATAG**GGCGCC**GCTGAGG 5
M.NgoAIII ”second”-sub[b]	5’ CAACTACT**CCGCGG**GATATC**C_T_GCGG**CGACTCC 3’<br>3’ GTTGATGA**GGCG_T_C**CTATAG**GGCGCC**GCTGAGG 5’
M.NgoAIII”third”-sub[b]	5’ CAACTACT**CCGCGG**GATATC**CCG_T_GG**CGACTCC 3’<br>3’ GTTGATGA**GG_T_GCC**CTATAG**GGCGCC**GCTGAGG 5’
M.NgoAIV-sub[b]	5’ CAACTACT**GCCGGC**GATATC**G_T_CGGC**CGACTCC 3’<br>3’ GTTGATGA**CGGC_T_G**CTATAG**CGGCCG**GCTGAGG 5’
M.NgoAVII-sub[b]	5’ CAACTACT**GCGGC**GATATC**G_T_GGC**CGACTCC 3’<br>3’ GTTGATGA**CGC_T_G**CTATAG**CGCCG**GCTGAGG 5’

M.NgoAORF302P-sub[b]	5’ CAACTACT**GCCGGT**GATATC**G*T*CGGT**CGACTCC 3’<br>3’ GTTGATGA**CGGC*T*A**CTATAG**CGGCCA**GCTGAGG 5’
M.NgoAORF1175P-sub[b]	5’ CAACTACT**CCGG**GATATC**C*T*GG**CGACTCC 3’<br>3’ GTTGATGA**GG*T*C**CTATAG**GGCC**GCTGAGG 5’
M.HhaI-sub[b]	5’ CAACTACT**GCGC**GATATC**G*T*GC**CGACTCC 3’<br>3’ GTTGATGA**CG*T*G**CTATAG**CGCG**GCTGAGG 5’
M.EcoKDcm-sub[b]	5’ CAACTACT**CCAGG**GATATC**C*T*AGG**CGACTCC 3’<br>3’ GTTGATGA**GGT*T*C**CTATAG**GGTCC**GCTGAGG 5’
TT[c]	5’ CAACTACT**GCCGTC**GATATC**G*T*CGGC**CGACTCC 3’<br>3’ GTTGATGA**CGGC*T*G**CTATAG**CTGCCG**GCTGAGG 5’

[a] Table includes only central part of DNA substrates; the whole sequence is given in Methods section.

[b] The bases in boldface are the recognition sequence for the various C5MTases, and underlined and italic T indicates the thymine which would arise as results deamination of m5C.

[c] The bases in boldface surround T:T mismatch.

Table S4. The mutations occurred in the *rpoB* gene in the gonococcal mutants with disrupted *vsr* genes.

Strain	Mutation [a]	Position of mutation in nucleotide sequence of *rpoB* gene	Result of mutation	% of all detected mutation
N. gonorrhoeae ngoAXIIIV::km	C→T<br>G→T<br>A→T	1646<br>1627<br>1958	S549F<br>D543Y<br>D653V	60%<br>26%<br>14%
N. gonorrhoeae ngoAXIVV::km	C→T<br>G→T<br>C→A	1772<br>1681<br>1646	P591L<br>G561C<br>S549Y	84%<br>8%<br>8%

[a] The table includes mutations detected in *rpoB* gene only in *N. gonorrhoeae* mutants.

Table S5. The activity of gonococcal Vsr endonucleases in the presence of the gonococcal MutL protein.

Table S5A.

	The activity of V.NgoAXIII endonuclease [a]		
substrate name (sequence)	in the presence of the $MutL_{Ngo}$ protein and ATP	in the presence of the $MutL_{Ngo}$ protein without ATP	in the absence of the $MutL_{Ngo}$ protein
M.NgoAI-sub[b] (AG**T**GCT)	0.0762[c] (↑103.9%)[ad]	0.0706 (↑89.0%)[ad]	0.0373
M.NgoAII-sub (GG**T**C)	0.0579 (↑62.6%)[a]	0.0495 (↑39.0%)[a]	0.0356

M.NgoAIII “first”-sub (**T**CGCGG)	0.0698 (↑87.2%)[a]	0.0643 (↑72.5%)[a]	0.0373
M.NgoAIII “second” –sub (C**T**GCGG)	0.0752 (↑107.1%)[a]	0.0660 (↑81.8%)[a]	0.0363
M.NgoAIII “third” -sub (CCG**T**GG)	0.0494 (↑87.1%)[a]	0.0429 (↑62.5%)[a]	0.0264
M.NgoAIV-sub (G**T**CGGC)	0.0487 (↑91.7%)[a]	0.0444 (↑74.8%)[a]	0.0254
M.NgoAVII-sub (G**T**GGC)	0.0664 (↑81.9%)[a]	0.0582 (↑59.4%)[a]	0.0365
M.NgoA1175P-sub (C**T**GG)	0.0408 (↑93.3%)[a]	0.0397 (↑88.1%)[a]	0.0211
M.HhaI-sub (G**T**GC)	0.0298 (↑86.5%)[a]	0.0261 (↑63.1%)[a]	0.0160
M.EcoKDcm-sub (C**T**AGG)	0.0394 (↑97.0%)[a]	0.0359 (↑79.5%)[a]	0.0200

Table S5B.

	The activity of V.NgoAXIV endonuclease [a]		
substrate name (sequence)	in the presence of the MutL$_{Ngo}$ protein and ATP	in the presence of the MutL$_{Ngo}$ protein without ATP	in the absence of the MutL$_{Ngo}$ protein
M.NgoAI-sub[b] (AG**T**GCT)	0.0343[c] (↑73.5%)[ad]	0.0322 (↑63.0%)[ad]	0.0198
M.NgoAIII "first"-sub (**T**CGCGG)	0.0313 (↑90.1%)[a]	0.0282 (↑71.2%)[a]	0.0165
M.NgoAIII "second" –sub (C**T**GCGG)	0.0810 (↑93.0%)[a]	0.0711 (↑69.3%)[a]	0.0420
M.NgoAIII "third" -sub (CCG**T**GG)	0.0430 (↑105.0%)[a]	0.0384 (↑82.8%)[a]	0.0210
M.NgoAIV-sub (G**T**CGGC)	0.0488 (↑88.0%)[a]	0.0425 (↑63.6%)[a]	0.0260
M.NgoAVII-sub (G**T**GGC)	0.0393 (↑87.2%)[a]	0.0303 (↑44.3%)[a]	0.0210
M.NgoA302P-sub (G**T**CGGT)	0.0799 (↑95.0%)[a]	0.0727 (↑77.4%)[a]	0.0410

M.NgoA1175P-sub (C**T**GG)	0.0924 (↑110.0%)[a]	0.0812 (↑84.6%)[a]	0.0440
M.HhaI-sub (G**T**GC)	0.0560 (↑100.0%)[a]	0.0492 (↑75.8%)[a]	0.0280

[a] The statistically significant differences (p value < 0.05).

[b] The sequence (5’ → 3’) recognized by the Vsr endonuclease is given in brackets, thymine mispaired with guanine is underlined and bolded.

[c] The activity of gonococcal Vsr endonucleases was determined by fitting the data to a first-order rate constant (k_{st}).

[d] The level of changes (%) of the activity of gonococcal Vsr endonuclease in the presence of $MutL_{Ngo}$ protein compared to activity Vsr enzyme alone.

↑ The increase the efficiency of the reaction catalyzed by gonococcal Vsr endonuclease.

Table S6. The activity of gonococcal Vsr endonucleases in the presence of MutL protein of *E. coli*.

Table S6A.

	The activity of V.NgoAXIII endonuclease [a]		
substrate (sequence)	in the presence of the $MutL_{E.coli}$ protein	in the absence of the $MutL_{E.coli}$ protein	level of changes (%)
M.NgoAI-sub[b]	0.0679[c]	0.0388	↑75.0% [a d]

(AG**T**GCT)			
M.NgoAII-sub (GG**T**C)	0.0655	0.0344	↑90.6%[a]
M.NgoAIII "first"-sub (**T**CGCGG)	0.0670	0.0336	↑99.5%[a]
M.NgoAIII "second" -sub (C**T**GCGG)	0.0685	0.0382	↑79.5%[a]
M.NgoAIII "third"-sub (CCG**T**GG)	0.0498	0.0274	↑81.9%[a]
M.NgoAIV-sub (G**T**CGGC)	0.0506	0.0254	↑99.4%[a]
M.NgoAVII-sub (G**T**GGC)	0.0650	0.0365	↑78.1%[a]
M.NgoA302P-sub (G**T**CGGT)	0.0913	0.0415	↑120.1%[a]
M.NgoA1175P-sub	0.0439	0.0221	↑98.7%[a]

(C**T**GG)			
M.HhaI-sub<br><br>(G**T**GC)	0.0652	0.0349	↑86.4%[a]
M.EcoKDcm-sub<br><br>(C**T**AGG)	0.0623	0.0325	↑91.6%[a]

Table S6B.

	The activity of V.NgoAXIV endonuclease [a]		
substrate<br><br>(sequence)	in the presence of the $MutL_{E.coli}$ protein	in the absence of the $MutL_{E.coli}$ protein	level of changes (%)
M.NgoAI-sub[b]<br><br>(AG**T**GCT)	0.0329[c]	0.0188	↑75.0% [a d]
M.NgoAIII<br><br>“first”-sub<br><br>(**T**CGCGG)	0.0289	0.0155	↑86.9%[a]
M.NgoAIII<br><br>“second”-sub<br><br>(C**T**GCGG)	0.0780	0.040	↑95.0%[a]
M.NgoAIII	0.0389	0.0197	↑96.7%[a]

"third"-sub (CCG**T**GG)			
M.NgoAIV-sub (G**T**CGGC)	0.0450	0.0250	↑80.0%[a]
M.NgoAVII-sub (G**T**GGC)	0.0340	0.0190	↑78.9%[a]
M.NgoA302P-sub (G**T**CGGT)	0.0786	0.0414	↑90.0%[a]
M.NgoA1175P-sub (C**T**GG)	0.0974	0.0497	↑98.7%[a]
M.HhaI-sub (G**T**GC)	0.0499	0.0290	↑72.3%[a]

[a] The statistically significant differences (p value < 0.05).

[b] The sequence (5' → 3') recognized by the Vsr endonuclease is given in brackets, thymine mispaired with guanine is underlined and bolded.

[c] The activity of gonococcal Vsr endonucleases was determined by fitting the data to a first-order rate constant (k_{st}).

[d] The level of changes (%) of the activity of gonococcal Vsr endonuclease in the presence of MutL$_{E.coli}$ protein compared to activity Vsr enzyme alone.

↑ The increase the efficiency of the reaction catalyzed by gonococcal Vsr endonuclease.

Table S7. The activity of gonococcal Vsr endonucleases in the presence of the gonococcal MutS protein.

Table S7A.

	The activity of V.NgoAXIII endonuclease [a]		
substrate (sequence)	in the presence of the MutS protein	in the absence of the MutS protein	level of changes (%)
M.NgoAI-sub[b] (AG**T**GCT)	0.0076[c]	0.037	↓79.4%[ad]
M.NgoAII-sub (GG**T**C)	0.0047	0.0356	↓86.6%[a]
M.NgoAIII “first”-sub (**T**CGCGG)	0.0045	0.0437	↓89.7%[a]
M.NgoAIII “second” -sub (C**T**GCGG)	0.0091	0.0363	↓74.9%[a]
M.NgoAIII “third”-sub (CCG**T**GG)	0.0032	0.0264	↓87.8%[a]

M.NgoAIV-sub (G**T**CGGC)	0.0036	0.0254	↓85.6%[a]
M.NgoAVII-sub (G**T**GGC)	0.0063	0.0365	↓82.7%[a]
M.NgoA302P-sub (G**T**CGGT)	0.0038	0.0405	↓90.6%[a]
M.NgoA1175P-sub (C**T**GG)	0.0076	0.0440	↓82.7%[a]
M.HhaI-sub (G**T**GC)	0.00246	0.0160	↓84.6%[a]
M.EcoKDcm-sub (C**T**AGG)	0.0046	0.0200	↓77.0%[a]

Table S7B.

	The activity of V.NgoAXIV endonuclease [a]		
substrate (sequence)	in the presence of the MutS protein	in the absence of the MutS protein	level of changes (%)
M.NgoAI-sub[b]	0.00722[c]	0.0198	↓63.5%[ad]

(AG**T**GCT)			
M.NgoAIII "first"-sub (**T**CGCGG)	0.0066	0.0165	↓60.0%[a]
M.NgoAIII "second"-sub (C**T**GCGG)	0.0155	0.0420	↓63.0%[a]
M.NgoAIII "third"-sub (CCG**T**GG)	0.0073	0.0210	↓65%[a]
M.NgoAIV-sub (G**T**CGGC)	0.0109	0.0260	↓58%[a]
M.NgoAVII-sub (G**T**GGC)	0.0110	0.0210	↓47.2%[a]
M.NgoA302P-sub (G**T**CGGT)	0.0143	0.0410	↓65%[a]
M.NgoA1175P-sub (C**T**GG)	0.011	0.0440	↓75%[a]
M.HhaI-sub	0.0103	0.0280	↓63%[a]

(G<u>**T**</u>GC)			

[a] The statistically significant differences (p value < 0.05).

[b] The sequence (5’ → 3’) recognized by the Vsr endonuclease is given in brackets, thymine mispaired with guanine is underlined and bolded.

[c] The activity of gonococcal Vsr endonucleases was determined by fitting the data to a first-order rate constant (k_{st}).

[d] The level of changes (%) of the activity of gonococcal Vsr endonuclease in the presence of MutS protein compared to activity Vsr enzyme alone.

↓The decrease of the efficiency of the reaction catalyzed by the gonococcal Vsr endonucleases.

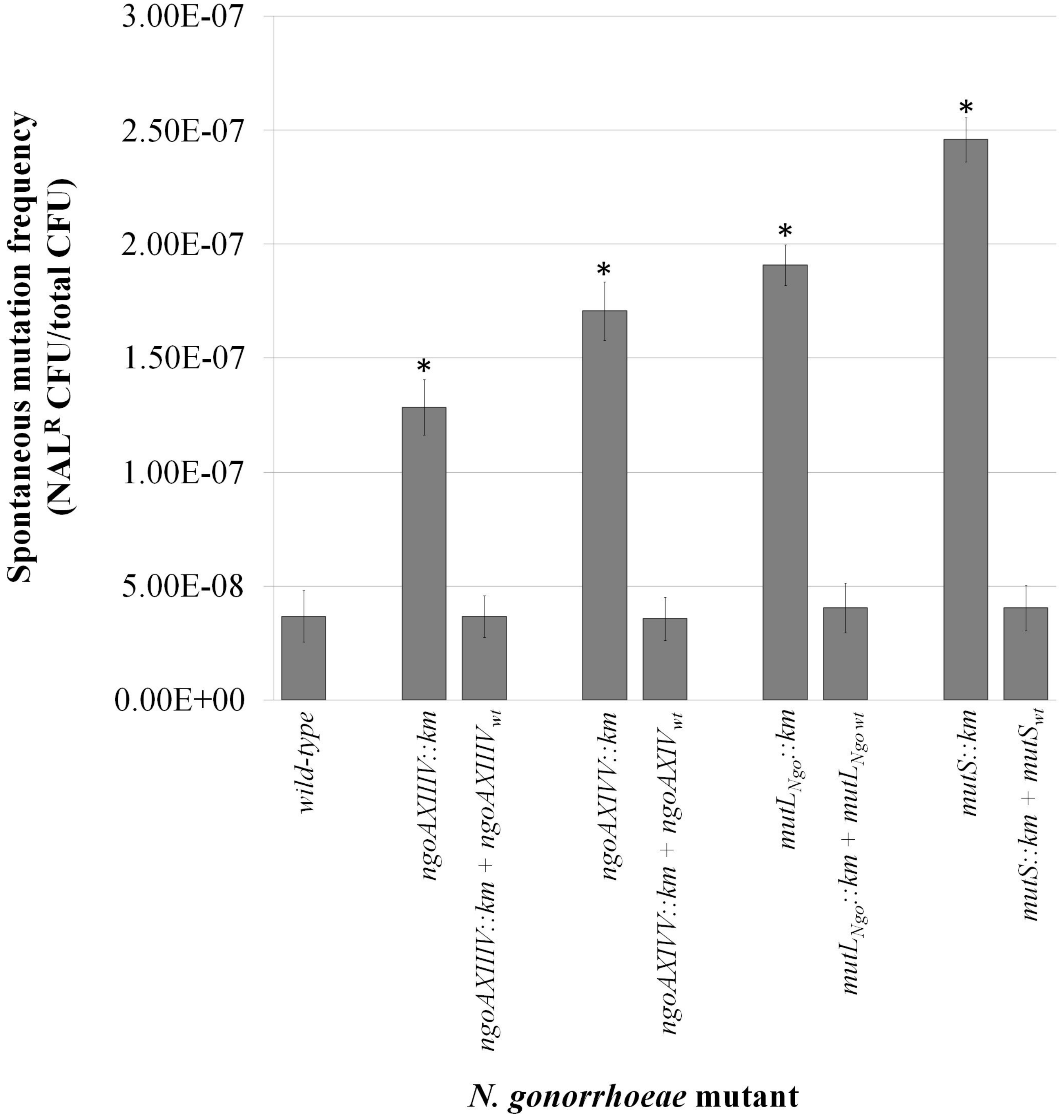

Fig. S1. Spontaneous mutation frequency in *N. gonorrhoeae* mutants with disrupted *ngoAXIIIV*, *ngoAXIVV*, *mutL*$_{Ngo}$ or *mutS* genes. A sample of liquid culture (0.1 ml, 10^8 cells) of each strain was plated on GC agar supplemented with nalidixic acid (NAL) or without antibiotics. After incubation at 37°C in 5% CO_2 for 48 h the colony numbers were counted and the frequency of spontaneous mutations was determined. Asterisks indicate statistically significant differences as was calculated using the Student's *t*-test (p value < 0.05).

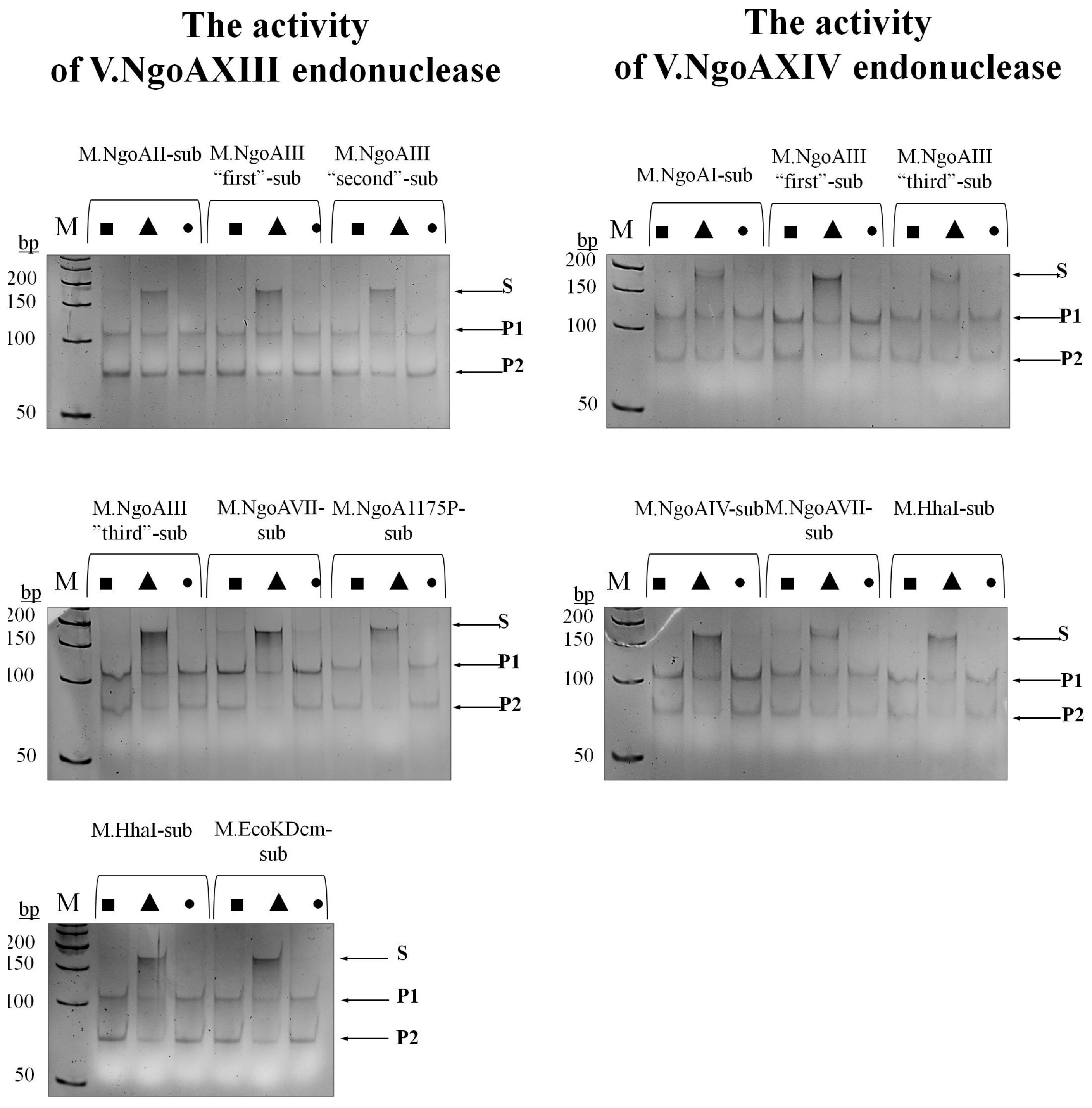

Fig. S2. The presence of the MutL$_{Ngo}$ protein decreases amount of Vsr endonuclease required to complete DNA digestion. Lanes marked by black square represent reactions carried out by Vsr endonuclease alone when molar ratio DNA:protein was 1:10; lanes marked by black triangle represent reactions carried out by Vsr endonuclease alone when molar ratio DNA:protein was 1:2; lanes marked by black circle represent reactions carried out by Vsr endonuclease in the presence of MutL$_{Ngo}$ when molar ratio DNA:protein was 1:2. Reactions were carried out using 0.15 μM substrate DNA, 0.3 μM or 1.5 μM Vsr endonuclease and where is indicated 0.6 μM MutL$_{Ngo}$ protein. M – marker GeneRuler 50 bp DNA Ladder (Thermo Scientific). Arrows indicate substrate and reaction products obtained after DNA cleavage by a given Vsr endonuclease. P1 and P2 reaction products; S – substrate DNA. The experiments were performed in triplicate and representative images are shown.

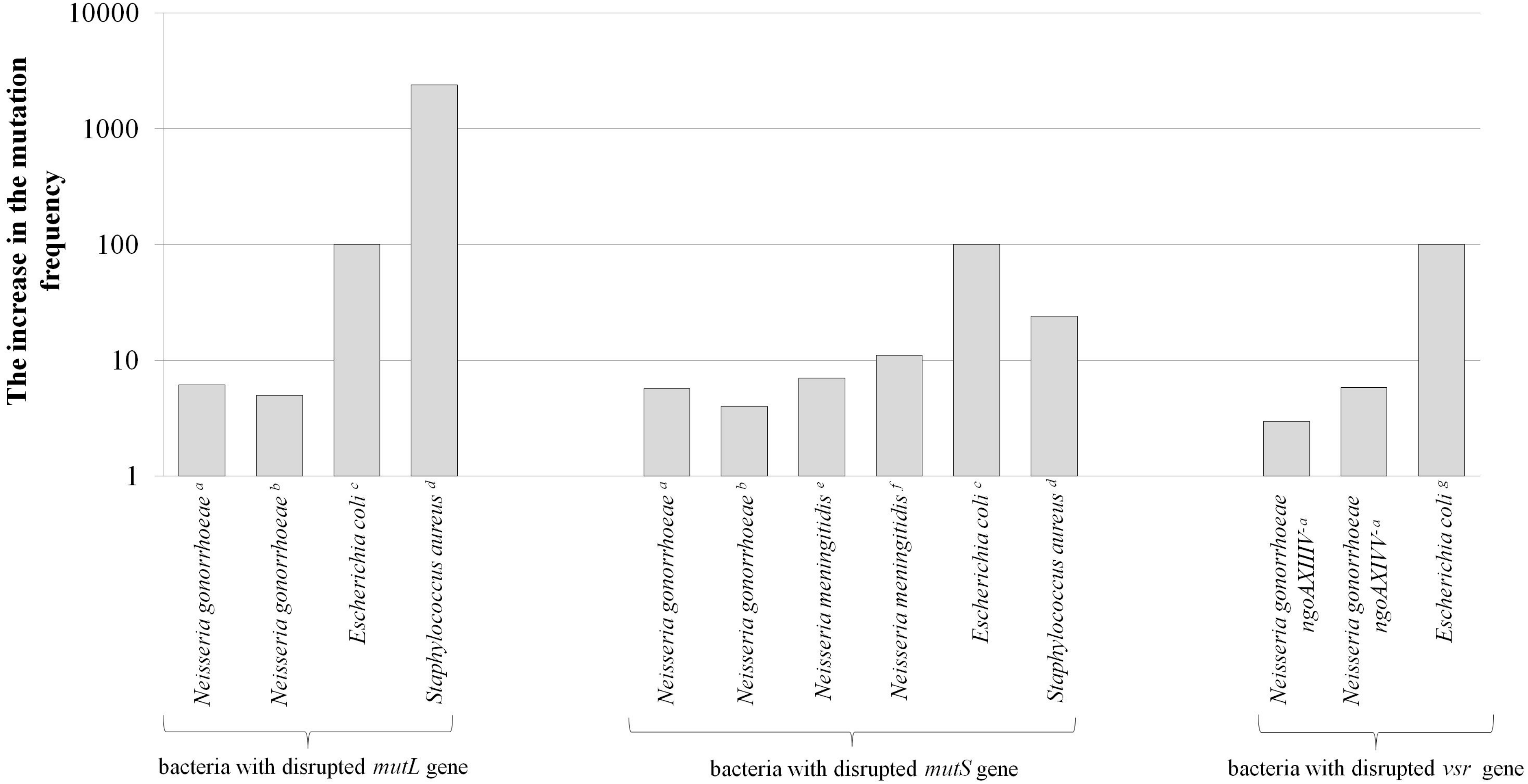

Fig. S3. Comparison of the increase in mutation frequency in different bacterial species with disrupted *mutL*, *mutS* or *vsr* genes. In each bacterial species, the frequency of mutations was compared to the wild type. [a] – the results obtained in this study; [b] – according to Criss *et al*. (2010); [c]- according to Wu and Marinus (1994); [d] - according to Prunier and Leclercq (2005); [e] - according to Davidsen *et al*. (2005), [f] – according to Martin *et al*. (2004); [g] – according to Bandaru *et al*. (1995).

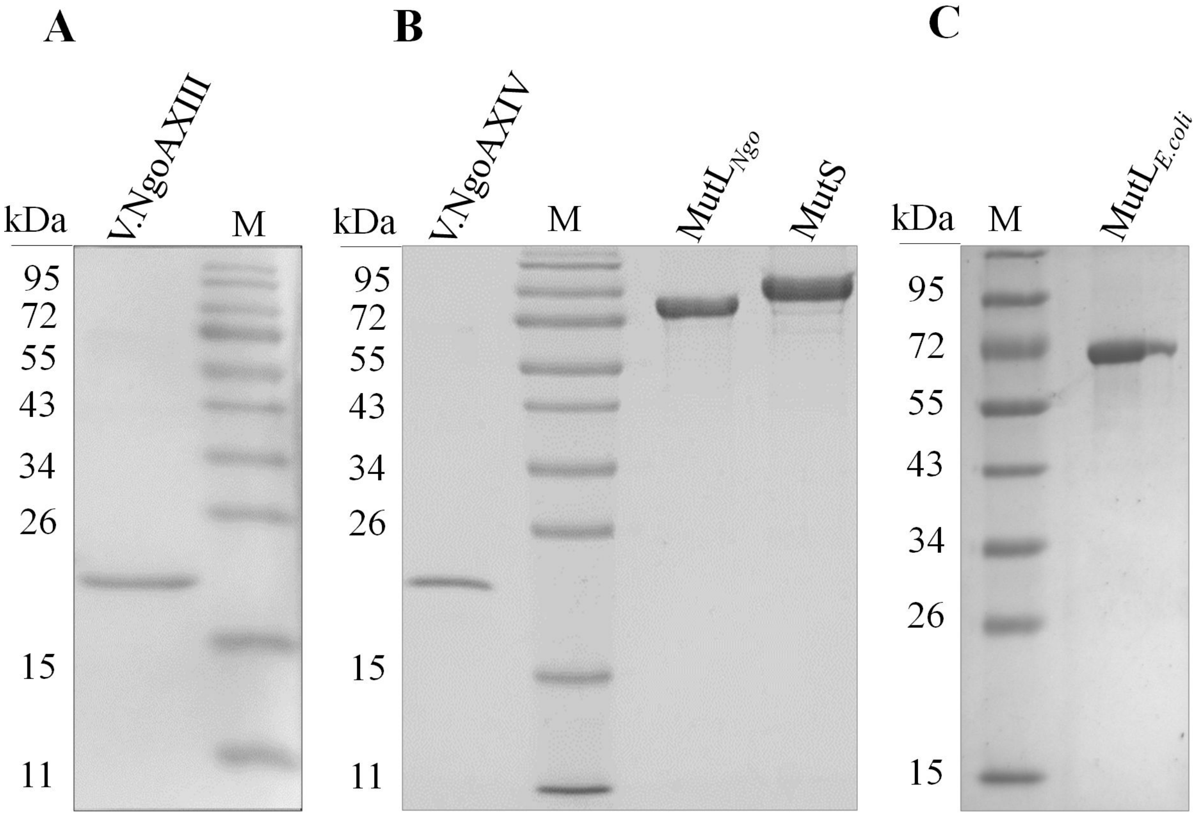

Fig. S4. Purification of (A) the V.NgoAXIII endonuclease (16 kDa), (B) V.NgoAXIV (18 kDa) endonuclease, $MutL_{Ngo}$ (74 kD) and MutS (97 kDa) protein of *N. gonorrhoeae* FA1090, (C) the MutL protein of *E. coli* K-12 (70 kDa). After purification by metal affinity chromatography, proteins were separated on a 10% or 12% SDS-PAGE gel and stained with Coomassie Brilliant blue R250. Lane M, PageRuler™ Prestained Protein Ladder (250, 130, 95, 72, 55, 43, 34, 26, 15 and 11 kDa) (Thermo Scientific).

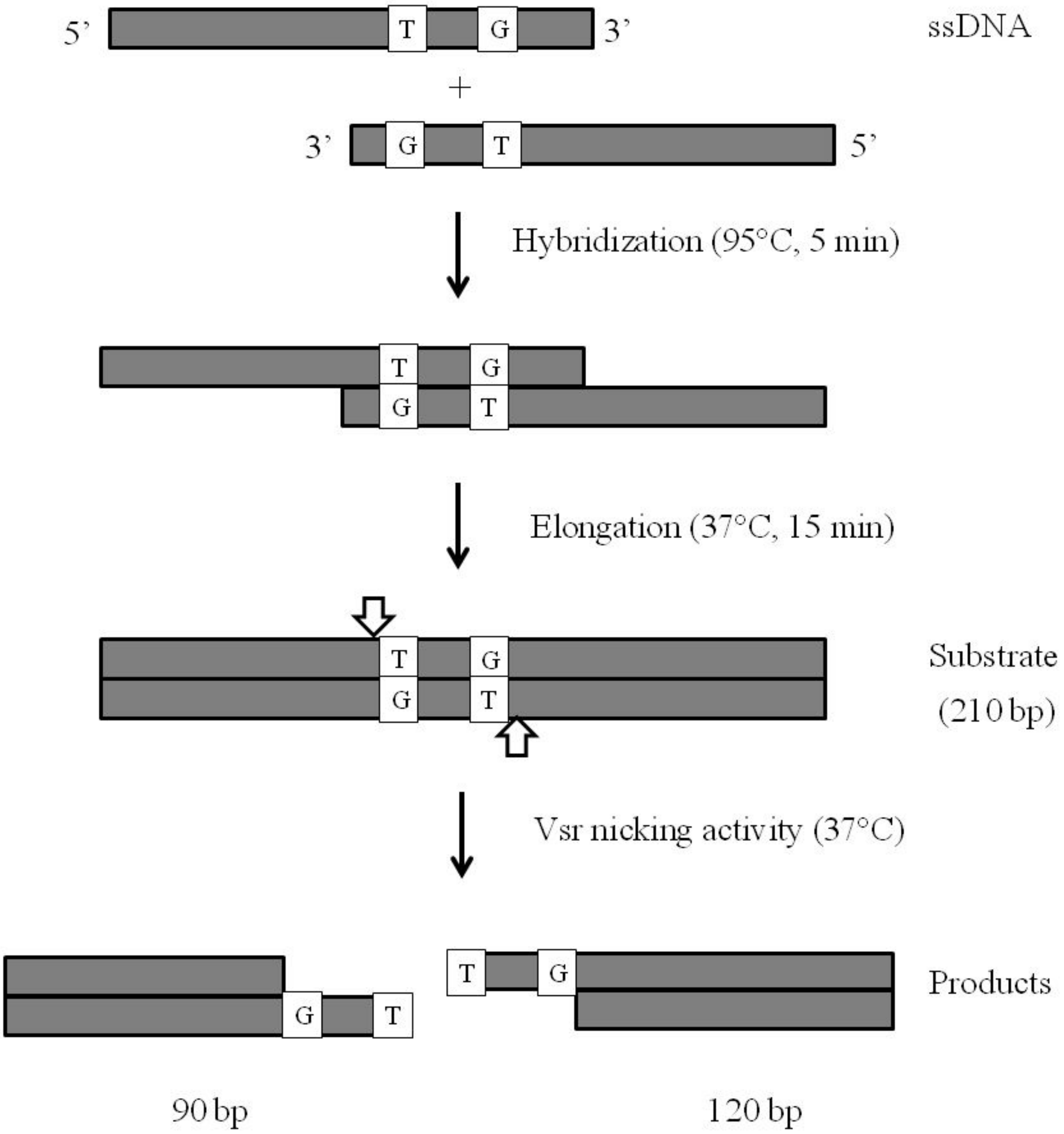

Fig. S5. Construction of DNA substrates containing two T:G mismatches and principle of an assay to demonstrate the activity of Vsr endonuclease. Each substrate DNA contains two sequences derived from the sequence modified by the individual m5C-MTases (in the substrate DNA, the modified cytosine was replaced by thymine). On the opposite strand in the sequence modified by m5C-MTase, at the site of the modified cytosine, there was a thymine mismatched to guanine. In this way, each substrate DNA (~210 bp) contained two T:G mismatches, which are indicated in white rectangles. The appearance of two fragments (~110 bp and ~80 bp) resulted from nicking the DNA with Vsr endonucleases on the 5' side of the two mismatched guanines to thymines. The sites of incision by Vsr endonuclease are indicated by wide white arrows. The details are included in the Methods section.

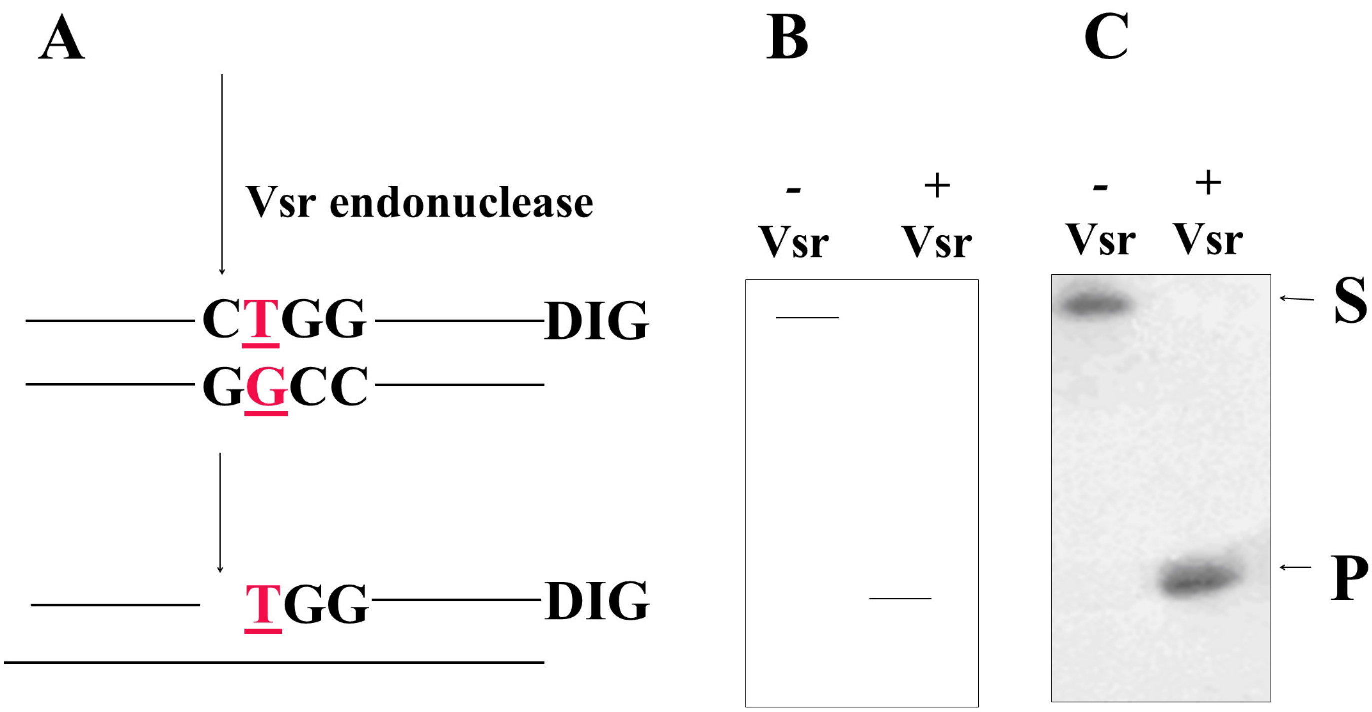

Fig. S6. (A, B) Construction of DNA substrates containing one T:G mismatch and principle of an assay to demonstrate the activity of Vsr endonuclease. Each substrate DNA (obtained by hybridization of two oligonucleotides) contains one sequence derived from the sequence modified by the individual m5C-MTases. In the substrate DNA, in one strand in the sequence modified by m5C-MTase, at the site of the modified cytosine, there was a thymine mismatched to guanine. In used assay, DNA strand with thymine was labeled with DIG Oligonucleotide 3'-End Labeling Kit according manufacturer recommendation's (Roche Diagnostic), followed hybridization of two oligonucleotides were carried out. The reaction products were separated in 15% polyacrylamide denaturing gels containing 8 M urea. DNA labelled with DIG (digoxigenin) was detected by chemiluminescence using Anti-Dioxigenin-AP antibodies and CSPD-ready–to-use reagent according manufacturer recommendation's (Roche Diagnostic). (C) Representative image of the activity of the studied Vsr endonucleases. On the image DIG-labelled DNA is visible. P - reaction product; S – substrate DNA.

The presented substrate DNA: 5' TACTTGGCTTATC**C<u>T</u>GG**AATCTGTCGCAG 3'/3' ATGAACCGAATAG**G<u>G</u>CC**TTAGACAGCGTC 5'). Other substrates were analogous. The only difference between individual substrate DNAs was the composition of the core sequence marked in boldface. The core sequence in each oligonucleotide pair corresponded to the sequence derived from known neisserial m5C-MTase recognition sequences.

REFERENCES:

1. Kwiatek A, Luczkiewicz M, Bandyra K, Stein DC, Piekarowicz A: **Neisseria gonorrhoeae FA1090 carries genes encoding two classes of Vsr endonucleases**. *J Bacteriol* 2010, **192**(15):3951-3960.
2. Criss AK, Bonney KM, Chang RA, Duffin PM, LeCuyer BE, Seifert HS: **Mismatch correction modulates mutation frequency and pilus phase and antigenic variation in Neisseria gonorrhoeae**. *J Bacteriol* 2010, **192**(1):316-325.
3. Wu TH, Marinus MG: **Dominant negative mutator mutations in the mutS gene of Escherichia coli**. *J Bacteriol* 1994, **176**(17):5393-5400.
4. Prunier AL, Leclercq R: **Role of mutS and mutL genes in hypermutability and recombination in Staphylococcus aureus**. *J Bacteriol* 2005, **187**(10):3455-3464.
5. Davidsen T, Bjørås M, Seeberg EC, Tønjum T: **Antimutator role of DNA glycosylase MutY in pathogenic Neisseria species**. *J Bacteriol* 2005, **187**(8):2801-2809.
6. Martin P, Sun L, Hood DW, Moxon ER: **Involvement of genes of genome maintenance in the regulation of phase variation frequencies in Neisseria meningitidis**. *Microbiology* 2004, **150**(Pt 9):3001-3012.
7. Bandaru B, Wyszynski M, Bhagwat AS: **HpaII methyltransferase is mutagenic in Escherichia coli**. *J Bacteriol* 1995, **177**(10):2950-2952.
